# 3,4-Dimethoxychalcone, a caloric restriction mimetic, enhances TFEB-mediated autophagy and alleviates pyroptosis and necroptosis after spinal cord injury

**DOI:** 10.7150/thno.78370

**Published:** 2023-01-01

**Authors:** Haojie Zhang, Wenfei Ni, Gaoxiang Yu, Yibo Geng, Junsheng Lou, Jianjun Qi, Yituo Chen, Feida Li, Hantao Ye, Haiwei Ma, Hui Xu, Luying Zhao, Yuepiao Cai, Xiangyang Wang, Huazi Xu, Jian Xiao, Kailiang Zhou

**Affiliations:** 1 Department of Orthopaedics, The Second Affiliated Hospital and Yuying Children's Hospital of Wenzhou Medical University, Wenzhou 325027, China; 2 Zhejiang Provincial Key Laboratory of Orthopaedics, Wenzhou 325027, China; 3 The Second Clinical Medical College of Wenzhou Medical University, Wenzhou 325027, China; 4 School of Pharmaceutical Sciences, Wenzhou Medical University, Wenzhou 325035, China; 5 Department of Orthopedic Surgery, The First Affiliated Hospital, Zhejiang University School of Medicine, Hangzhou 310003, China; 6 Department of Clinical Laboratory, The First Affiliated Hospital of Wannan Medical College, Wuhu 241001, China; 7 Department of Gastroenterology, The First Affiliated Hospital of Wenzhou Medical University, Wenzhou 325015, China

**Keywords:** 3, 4-dimethoxychalcone, spinal cord injury, TFEB, autophagy, pyroptosis, necroptosis

## Abstract

**Background:** Caloric restriction mimetics (CRMs) mimic the favourable effects of caloric restriction (CR) and have been shown to have therapeutic effects in neuroinflammatory disease. However, whether CRMs improve the functional recovery from spinal cord injury (SCI) and the underlying mechanism of action remain unclear. In this study, we used the CRMs 3,4-dimethoxychalcone (3,4-DC) to evaluate the therapeutic value of CRMs for SCI.

**Methods:** HE, Masson and Nissl staining; footprint analysis; and the Basso mouse scale were used to determine the functional recovery from SCI after 3,4-DC treatment. RNA sequencing was used to identify the mechanisms of 3,4-DC in SCI. Western blotting, qPCR and immunofluorescence were used to detect the levels of pyroptosis, necroptosis, autophagy and the AMPK-TRPML1-calcineurin signalling pathway. We employed a dual-luciferase reporter assay *in vitro* and applied AAV vectors to inhibit TFEB *in vivo* to explore the mechanism of 3,4-DC.

**Results:** 3,4-DC reduced glial scar area and motor neuron death and improved functional recovery after SCI. RNA-sequencing results indicated that oxidative stress, pyroptosis, necroptosis, and autophagy may be involved in the ability of 3,4-DC to improve functional recovery. Furthermore, 3,4-DC inhibited pyroptosis and necroptosis by enhancing autophagy. We also found that 3,4-DC enhances autophagy by promoting TFEB activity. A decrease in the TFEB level abolished the protective effect of 3,4-DC. In addition, 3,4-DC partially regulated TFEB activity through the AMPK-TRPML1-calcineurin signalling pathway.

**Conclusions:** 3,4-DC promotes functional recovery by upregulating TFEB-mediated autophagy and inhibiting pyroptosis and necroptosis after SCI, which may have potential clinical application value.

## Introduction

Spinal cord injury (SCI) is a highly disabling and catastrophic illness 1 SCI often leads to permanent paralysis and various motor, sensory and autonomic nervous system dysfunctions, which seriously affect quality of life and life expectancy 2. However, currently, there are no particularly effective treatments for SCI. The pathophysiological process of SCI is separated into two phases: primary injury and secondary injury. After the initial short-term, direct mechanical damage to the tissues, a long secondary injury follows. The secondary injury characteristics mainly include a series of reactions, such as neuroinflammation, ischaemia, oxidative stress, and neuronal death 3, 4. Since the primary injury cannot be prevented, current treatments mainly focus on intervention of secondary SCI 5. Current evidence indicates that inhibition of neuroinflammation and cell death during secondary injury is vital for treating SCI 6.

Cell death and inflammation are closely related 7. Pyroptosis is a recently discovered form of proinflammatory cell death. Pyroptosis occurs in a variety of tissues, including the spinal cord and brain, and is driven by inflammasomes 8. Inflammasomes are typically composed of NOD-like receptor protein (NLRP) 1 and NLRP3 promoter receptors, the adaptor protein apoptosis-related speck-like protein that contains a caspase-recruitment domain (ASC), and precursor caspase-1 9. Activation of the inflammasome complex triggers the lysis of pro-caspase-1, produces active caspase-1 p10/p20 tetramers, and induces transition of the proinflammatory cell factors interleukin (IL)-1β and IL-18 from their immature forms to their secreted active forms 10. Activated caspase-1 is capable of cleaving the gasdermin-D (GSDMD) protein to form bioactive GSDMD-N. Finally, GSDMD-N stimulates cell membrane perforation, which causes the release of inflammatory factors in the cell and triggers a series of inflammatory reactions 11. According to recent studies, the activation of cytoplasmic inflammasome complexes leading to pyroptosis is an essential step in the neuroinflammation related to secondary SCI damage 12. Abundant evidence suggests that microglia are important mediators of innate immune responses after central nervous system (CNS) injury, including SCI, and are the main cells in the CNS that undergo pyroptosis 13. After SCI, microglia are activated, and the expression of the NLRP3 inflammasome mediated by a large number of signalling cascades increases, causing pyroptosis in microglia and neurons and leading to aggravation of secondary injury 12, 13. Therefore, pyroptosis of microglia and neurons might be vital in the secondary stage of SCI.

Necroptosis is similar to pyroptosis, both of which are inflammatory cell death pathways 14. Mechanistically, the activation of TNF receptor 1 (TNFR1) under stress conditions leads to the activation of RIPK1 and RIPK3, which then assemble into a multiprotein complex containing the RIPK1/RIPK3 necrosome 15. Subsequently, necrosomes promote phosphorylation of the downstream molecule MLKL, and p-MLKL is ultimately oligomerized and translocated to the plasma membrane, increasing membrane permeability to induce cell death 16. Studies have also reported an important inhibitory role of caspase-8 in necroptosis 17. Necroptosis has been recognized as a key form of programmed cell death after SCI. Due to the rapid accumulation of reactive oxygen species and inflammatory markers, secondary SCI may induce necroptosis within hours 18. Recent studies have confirmed the existence of necroptosis mainly in neurons but also in microglia following SCI, which contributes to early neuronal damage after SCI in adult mice 19, 20. Therefore, inhibiting the occurrence and development of necroptosis in neurons is vital for treating SCI.

Autophagy is a lysosomal degradation process used to degrade damaged organelles, protein aggregates and cytoplasmic proteins 21. In CNS diseases, autophagy-induced neuroprotection is activated in both neurons and microglia 22, 23. Autophagy is vital for endocellular “refreshing”, and this effect on homeostasis is particularly pivotal for ensuring the health of terminally differentiated cells, such as neurons 24. Therefore, many studies suggest that autophagy plays an important role in neurons 25, 26. In addition, the induction of autophagy simultaneously inhibits the development of pyroptosis and necroptosis 27. Transcription factor EB (TFEB), which is a component of the microphthalmia transcription factor family, is considered a master modulator of autophagy activity and lysosome biogenesis 28. In other words, autophagy is strictly dependent on the regulation of lysosomal function by TFEB. Importantly, the impaired autophagy-lysosomal pathway is a component of the secondary damage after SCI 29, 30. As shown in our previous study, TFEB-mediated restoration of autophagy exerts a positive effect in the treatment of SCI 31. Furthermore, in neurodegenerative diseases, such as Alzheimer's and Parkinson's diseases, the induction of TFEB-mediated autophagy attenuates oxidative stress-induced neuronal death and plays an important role in improving the living environment of neurons 28. Therefore, approaches that regulate programmed cell death by enhancing the TFEB-mediated autophagy-lysosomal pathway may be effective strategies to increase neuronal survival and promote functional recovery after SCI.

Dietary restrictions, including calorie restriction (CR) and intermittent fasting, have been shown to have significant health-promoting and life-extending effects 32. Intermittent fasting has been shown to have positive effects in the treatment of SCI in rats but lacks efficacy in mice 33, 34. This may be due to the reduced ketogenic response in mice after intermittent fasting compared to that in rats 34. Although the health-promoting effects of CR are indisputable, long-term adherence greatly reduces quality of life. Therefore, caloric restriction mimetics (CRMs) were designed to simulate the favourable effects of CR without decreasing food consumption. CRMs are natural or synthetic compounds that mimic calorie restriction by reducing protein acetylation to induce protective autophagy 35. In recent years, various CRMs, such as resveratrol, metformin, and rapamycin, have been discovered and validated as treatments for SCI 36-38. However, the mechanistic basis of the neuroprotective effect of CRMs remains unclear. Notably, 3,4-dimethoxychalcone (3,4-DC), as a candidate CRMs, induces TFEB nuclear translocation and lysosomal biogenesis in the absence of toxicity, mediates an autophagy-dependent cardioprotective effect against ischaemic injury, and improves the efficacy of anticancer chemotherapy* in vivo* 39. Therefore, we used 3,4-DC to simulate the caloric restriction effect of CRMs and evaluated the therapeutic effects of CRMs in SCI. Our preliminary study involving RNA-sequencing and Gene ontology (GO) analyses revealed that 3,4-DC treatment may inhibit pyroptosis and necroptosis and upregulate autophagy and calcium signalling pathways. Furthermore, the AMP-activated protein kinase (AMPK)-transient receptor potential mucolipin 1 (TRPML1)-calcineurin signalling pathway regulates autophagy activity by modulating TFEB 40. Here, we established a contusion SCI model in mice to verify the therapeutic effect of 3,4-DC. Specifically, we attempted to determine (1) whether 3,4-DC exerts a neuroprotective effect in SCI; (2) whether pyroptosis, necroptosis and TFEB-mediated autophagy are involved in the mechanism by which 3,4-DC treatment alleviates SCI; and (3) whether TFEB activity is regulated by AMPK-TRPML1-calcineurin signalling.

## Methods

### Animals

Due to the shorter urethra of female animals, artificial voiding to prevent urine retention after SCI is easier; thus, female animals are often used in experimental SCI studies 41. Healthy adult C57BL/6J mice (females, 6-8 weeks old, mean body weight of 20-25 g) were acquired from the Experimental Animal Center (no. SCXK [ZJ] 2015-0001) at our university (Zhejiang, PRC). All mice were housed under normal conditions (21-25 °C, 12-h light/dark period, humidity: 50-60%) and allowed to eat and drink freely.

### Ethics statement

All experiments involving animals were conducted according to the ethical policies and procedures approved by the ethics committee of Wenzhou Medical University, China (Approval no. wydw 2017‒0096).

### Reagents and antibodies

3,4-Dimethoxychalcone (C17H16O3, HPLC ≥ 95%, cat# ZES-1294) was purchased from Extrasynthese (Genay, France). 3-Methyladenine (3MA) (C6H7N5, HPLC ≥ 99% purity, cat# M9281) was obtained from Sigma‒Aldrich (St. Louis, MO, USA). Compound C (C24H25N5O; purity ≥ 98.14%, cat# 866405-64-3), MHY1485 (C17H21N7O4; purity ≥ 99.86%, cat# HY-B0795), tacrolimus (C44H69NO12; purity ≥ 99.93%, cat# HY-13756), and gentamicin sulphate (C24H55N7O11S3; cat# HY-A0276) were purchased from Med Chem Express (Monmouth Junction, NJ, USA). The haematoxylin-eosin (HE) staining kit (cat# G1120), Masson staining kit (cat# G1340), and Nissl staining kit (cat# G1430) were acquired from Solarbio Science & Technologies (Beijing, PRC). AAV-TFEB shRNA (serotype 9, sequence: *CCAAGAAGGATCTGGACTT,* without a fluorescent reporter gene) was developed by Shanghai GeneChem Chemical Technologies (Shanghai, PRC). Similarly, a scramble shRNA control was constructed (sequence: *CGCTGAGTACTTCGAAATGTC*). AAV-TFEB shRNA and AAV-scrambled shRNA titres were detected via quantitative PCR to be 4.97E+12 vg/ml and 8.14E+12 vg/ml, respectively. A BCA kit (cat# 23227) and NE-PER™ nuclear and cytoplasm extraction reagents (cat# 78835) were purchased from Thermo Fisher Scientific (Rockford, IL, USA). Aqueous mounting medium containing DAPI and Fluoroshield (cat# ab104139) was purchased from Abcam (Cambridge, UK). A detailed list of primary antibodies is available in Table S1.

### Contusion SCI model, treatment and groups

Before surgery, every animal was anaesthetized via an intraperitoneal injection of 1% (w/v) pentobarbital sodium (50 mg/kg). A normal laminectomy was performed at the level of T9-T10 to expose the dorsum cord surface without damaging the dura, and a spinal cord impactor (W.M. KECK+, USA) was utilized to create injuries by dropping a rod (5 g) onto the spinal cord from a height of 3 cm. After injury, the muscles and skin were stitched in layers with 4-0 silk. Mice assigned to the Sham group were subjected to laminectomy alone at the same level. During recovery from anaesthesia, the mice were placed in a temperature-controlled room until thermoregulation was restored. The bladder was emptied by manually applying abdominal pressure three times a day after SCI until urination functions were restored. Antibiotic (gentamicin sulphate, 30 mg/kg) was administered via intraperitoneal injection once a day for 3 days post-surgery.

To record the dose‒response chart, we prepared 30 C57BL/6J mice separately. According to the dosage (mg/kg/day; 0, 100, 150, 200, 250), the mice were randomly divided into five groups with six mice in each group. Except for RNA sequencing (*n* = 3, where “*n*” represents the number of biological replicates), every assay had an identical group size (*n* = 6). The C57BL/6J mice (*n =* 276) were stochastically divided into fifteen groups: Sham (*n =* 30), SCI (*n =* 33), SCI+3,4-DC (200 mg/kg) (*n =* 33), SCI+3MA (*n =* 24), SCI+3,4-DC/3MA (*n =* 24), SCI+3,4-DC/scrambled shRNA (*n =* 24), SCI+3,4-DC/TFEB shRNA (*n =* 24), SCI+Compound C (CC) (*n =* 6), SCI+3,4-DC/Compound C (*n =* 6), SCI+MHY1485 (*n =* 6), SCI+3,4-DC/MHY1485 (*n =* 6), SCI+tacrolimus (*n =* 6), SCI+3,4-DC/tacrolimus (*n =* 6), SCI+3,4-DC (100 mg/kg) (*n =* 6), SCI+3,4-DC (150 mg/kg) (*n =* 6), SCI+3,4-DC (250 mg/kg) (*n =* 6), and Sham+3,4-DC (200 mg/kg) (*n =* 30). The mice in the Sham group underwent laminectomy without SCI. Mice in the SCI+3,4-DC group received an intraperitoneal injection of 3,4-DC dissolved in corn oil at a 200 mg/kg dose once daily for half an hour before surgery and 3 days after SCI surgery. The Sham and SCI groups were administered intraperitoneal injection of an equal volume of corn oil. After surgery, the SCI+3,4-DC/3MA group received an intraperitoneal injection of 15 mg/kg 3MA dissolved in saline 0.5 h before the 3,4-DC injection each day. The SCI+3,4-DC/Compound C group, the SCI+3,4-DC/MHY1485 group, and the SCI+3,4-DC/tacrolimus groups were intraperitoneally injected with Compound C dissolved in saline (1.5 mg/kg), MHY1485 dissolved in DMSO (2 mg/kg), and tacrolimus dissolved in DMSO (1 mg/kg), respectively, half an hour before the 3,4-DC injection. The SCI+MHY1485 group and SCI+tacrolimus group were injected with the same dose of MHY1485 and tacrolimus, respectively. In the SCI+3,4-DC/TFEB shRNA group, two weeks before surgery, after the intraperitoneal injection of 1% pentobarbital sodium, a normal laminectomy was performed at the T9-T10 level, microsyringes were inserted into the spinal cord at a 45° angle, and 8 sites were injected with 2 μL of viral vectors in PBS per site at a speed of 0.2 μlμL/min, while the SCI+3,4-DC/scrambled shRNA group was injected with an equal volume of the AAV vector. After the injection and suturing were completed, the mice did not develop hindlimb paralysis or paresis after injection. Table S2 shows the group distribution and associated procedures and treatments. The mice were sacrificed by administration of an overdose of pentobarbital sodium, and histological specimens were obtained for relevant assays at days 3, 14 and 28.

### Functional behavioural evaluation

Hind limb motor function was evaluated by determining the Basso Mouse Scale (BMS) score and the BMS subscore and performing a footprint analysis at different time points. BMS score ranged between 0 and 9 (0 = panplegia to 9 = normal motor function) based on hindlimb joint movement and coordination 42. The footprint analysis compared the movements of mice in different groups. The forelimbs and hind limbs were dyed blue and red, respectively. Each mouse was evaluated by two researchers who were unaware of the treatment.

### Tissue section preparation and HE, Nissl and Masson staining

On days 14 and 28, the mice were deeply anaesthetized again and infused with phosphate-buffered normal saline (PBS, pH 7.4) through the heart; then, 4% (w/v) paraformaldehyde (PFA) was added to the PBS, and the animals were perfused. Next, the rostral spinal cord segments (1 mm in length, 4 mm from the epicentre) and the whole segment (10 mm long, with the epicentre in the centre) were excised and fixed with 4% (w/v) PFA for 24 h. The tissues were successively dehydrated in a gradient of ethanol solutions. The treated tissue was then embedded in paraffin in the appropriate direction. The paraffin-embedded spinal cord was sliced into 5-micrometer sections with a microtome and mounted on a gel-coated slide. Transverse and longitudinal sections were histopathologically examined using HE staining. Transverse sections were soaked in 1% cresyl violet acetate for Nissl staining according to the supplier's specifications. Nissl-positive cells were visualized to define the neurons in the anterior horns. For Masson's trichrome staining, longitudinal sections were incubated with a mixture of 10% potassium dichromate and 10% trichloroacetic acid, and the nuclei were dyed with haematoxylin. Afterwards, the sections were subjected to differentiation in hydrochloric acid and ethanol, reduced to blue with weak NH_3_, and stained with a Masson solution. Eventually, bright-field images were acquired using a light microscope (Olympus, Japan). Thresholding method in ImageJ to quantify the Masson-stained lesion area (blue).

### Western blotting (WB)

The animals were euthanized with an overdose of sodium pentobarbital on day 3 after SCI, and 5 mm spinal cord tissue samples were acquired from around the injury centre in the SCI groups and from the same area in the control group and immediately stored at -80 °C. Tissue samples were treated with RIPA lysis solution (Beyotime) containing a protease inhibitor cocktail (Sigma‒Aldrich) and phosphatase inhibitor cocktail III (Sigma‒Aldrich). A tissue homogenizer (OMNI Prep multisample homogenizer) was used to homogenize each sample for 60 s/pulse with a gap of 300 s for 3-5 consecutive pulses. Then, the protein concentration was determined using a Pierce BCA testing kit (Thermo Fisher Scientific). Cytoplasmic and nuclear proteins were extracted using a NE-PER™ kit, as previously described 43. Equal amounts of proteins (60 μg) were separated by electrophoresis on 12% (w/v) gels and then transferred to PVDF membranes (Millipore). After blocking with 5% (w/v) skim milk at ambient temperature for 2 h, the membranes were incubated overnight with the appropriate primary antibodies at 4 °C. Information on the dilution concentrations of the antibodies is listed in Table S1. The membranes were then incubated with enzyme-conjugated IgG secondary antibody at ambient temperature for 120 min, and images of the bands on the membranes were acquired using the ECL Plus Reagent Tool. Eventually, the band intensity was quantified using Image Lab 3.0 software (Bio-Rad).

### Immunofluorescence staining

The transverse and longitudinal sections of spinal cord tissue were prepared at 5-micrometer thickness and subjected to immunofluorescence staining, as described above. The tissue samples from all groups were deparaffinized, rehydrated, washed, placed in a 10.2 mM sodium citrate buffer solution and incubated at 95 °C for 20 min. After 10 min of permeabilization with 0.1% (v/v) PBS-Triton X-100, the sections were subjected to blocking with 10% (v/v) goat serum albumin in PBS (1 h) and incubated with primary antibodies overnight at 4 °C. Information regarding the dilution concentrations of antibodies is presented in Table S1. Finally, sections were incubated with secondary antibody at 37 °C for 1 h and counterstained with DAPI solution.

### Immunofluorescence quantification

Longitudinal sections of the spinal cord were immunofluorescently stained for MAP2 and GFAP. The lesion site was photographed using a Zeiss LSM 800 confocal microscope. Image acquisition and processing were performed with Zen Blue software (Zeiss), and image presentation was performed using ImageJ software (Version 1.52a). All images of transverse sections were captured 0.5-1 mm rostral to the lesion site, and imaging results were assessed using a fluorescence microscope (Olympus, Japan) in 6 random acquisition areas in the anterior horn of 3 random sections from each specimen. The integrated density of caspase-1, GSDMD-N, RIPK1, RIPK3 and p62 in each neuron was calculated using ImageJ software. The numbers of LC3 puncta in neurons, caspase-1-positive microglia (per 0.1 mm^2^), and GSDMD-N-positive microglia (per 0.1 mm^2^) were determined manually in a double-blind manner.

### Dual-luciferase reporter assay

The cloned CTSD gene containing 200 bp of the coordinated lysosomal expression and regulation (CLEAR)-box sequence (gccacgtgag, the specific sequence region of TFEB binding) was inserted into the luciferase vector pGL3-basic (Promega, Madison, WI) and named CLEAR-box+Empty. The CLEAR-box sequence was mutated (TAACTAGTTA) and named CLEAR-box-mut+Empty. The recombinant TFEB sequence was inserted into pcDNA3.1 and named CLEAR-box+TFEB and CLEAR-box-mut+TFEB. A mixture of plasmids (plasmid ratio CLEAR-Box:TFEB:TK = 5:5:1, total plasmid amount: 2 μg) and 2 μL of Lipofectamine 2000 transfection reagent (Invitrogen, Carlsbad, CA) were added to the medium of 293 cells for cotransfection. Then, 15, 30, or 60 μM 3,4-DC was added 24 h after transfection, and the samples were named CLEAR-box+TFEB+15 μM, CLEAR-box+TFEB+30 μM, and CLEAR-box+TFEB+60 μM, respectively. The cells were collected 48 h after transfection, and a luciferase detection kit (Beyotime, RG027) was used to detect firefly luciferase and Renilla luciferase intensities. The relative luciferase intensities were calculated.

### RNA sequencing and functional enrichment analyses

Tissue was collected 3 days after SCI, and total RNA was extracted with TRIzol reagent according to the supplier's specifications. RNA purity and concentration were assessed using a NanoDrop 2000 spectrophotometer (Thermo Scientific, USA). RNA integrity was evaluated with an Agilent 2100 biological analyser (Agilent Technology, USA). Then, libraries were constructed using a TruSeq Stranded mRNA LT Sample Prep Kit (Illumina, San Diego, CA, USA) according to the manufacturer's instructions. Transcriptome sequencing and analysis were conducted by OE Biotech Co., Ltd. (Shanghai, China). Afterwards, libraries were constructed by sequencing with an Illumina HiSeq X Ten system, and 125 bp/150 bp paired-end reads were produced. The raw data were processed using Trimmomatic. Data that contained poly-N reads and low-quality reads were discarded, and clean reads were preserved for subsequent assays. The clean reads were mapped to the murine genome (GRCm38.p6) via HISAT2. Fragments per kilobase of transcript per million mapped reads (FPKM) values were produced for every gene using Cufflinks, and the read counts of every gene were acquired using HTSeqcount. Differential expression analyses were conducted via the DESeq (2012) package for R. P < 0.05 and a fold change > 2 or < 0.5 were considered the liminal values. Layer clustering analyses of differentially expressed genes (DEGs) were performed to identify the genetic expression features of different groups and specimens. A GO enrichment analysis of DEGs was completed using R based on the hypergeometric distribution.

### ELISA

Spinal cord tissues were homogenized in PBS and repeatedly frozen in liquid nitrogen and thawed. The homogenate was centrifuged for 10 min at 10,000 × g and 4 °C, and the tissue supernatant was collected for further testing. The levels of cleaved IL-1β in spinal cord lesions were detected using an enzyme-linked immunosorbent assay (ELISA) kit according to the manufacturer's protocol (Boyun Biotechnology, Shanghai, China). Finally, for quantification of cleaved IL-1β, the OD of the samples was determined using a microplate reader at 550 nm with a correction wavelength of 450 nm.

### Quantitative PCR (qPCR)

Total RNA was extracted from the spinal cord using TRIzol reagent according to the manufacturer's instructions. Quantitative analyses were completed via a two-step reaction procedure: reverse transcription (RT) and PCR. Every RT reaction comprised 0.5 μg of RNA, 2 μL of 5×*TransScript* All-in-one SuperMix for qPCR and 0.5 μL of gDNA remover in an overall volume of 10 μL. The reaction was conducted using a GeneAmp® PCR System 9700 (Applied Biological Systems, USA) at 42 °C for 15 min, followed by 85 °C for 5 s. The 10 μL RT reaction mixture was then diluted 10-fold in nuclease-free water and incubated at -20 °C. Real-time PCR was conducted using a LightCycler® 480 II Real-time PCR System (Roche, Switzerland) with a 10 μL PCR mix that included 1 μL of cDNAs, 5 μL of 2×*PerfectStart*^TM^ Green qPCR SuperMix, 0.2 μL of forward primer, 0.2 μL of reverse primer and 3.6 μL of nuclease-free water. The reaction process was incubated in a 384-well optic plate (Roche) at 94 °C for 0.5 min, followed by 45 cycles at 94 °C for 5 s and 60 °C for 30 s. All specimens were analysed three times. After the PCR cycles, melting curve analyses were performed to verify the specific generation of the anticipated PCR products. The primer sequences were developed in the lab and synthesized by TsingKe Biological Technology based on the mRNA sequences acquired from the NCBI database and are listed in Table S3. Finally, the mRNA expression levels were normalized to *β-actin* expression and computed using the 2^-ΔΔCt^ approach.

### Statistics

Statistical analyses were performed with GraphPad Prism Software, version 8.0.1 for Windows (San Diego, CA). All data are presented as the means ± standard errors of the means (SEMs). All data are the results of statistical analysis of measured parameters. Comparisons between two independent groups were performed using a two-tailed, unpaired t test. Two-way analysis of variance (ANOVA) with Tukey's multiple comparisons test was used to analyse differences among three or more groups when the data were normally distributed, and nonparametric Mann-Whitney U tests were used for groups if the data were not normally distributed. *P < 0.05, **P < 0.01, and ***P < 0.001 indicate significant differences.

## Results

### 3,4-DC improves functional restoration after SCI

**Figure 1:**
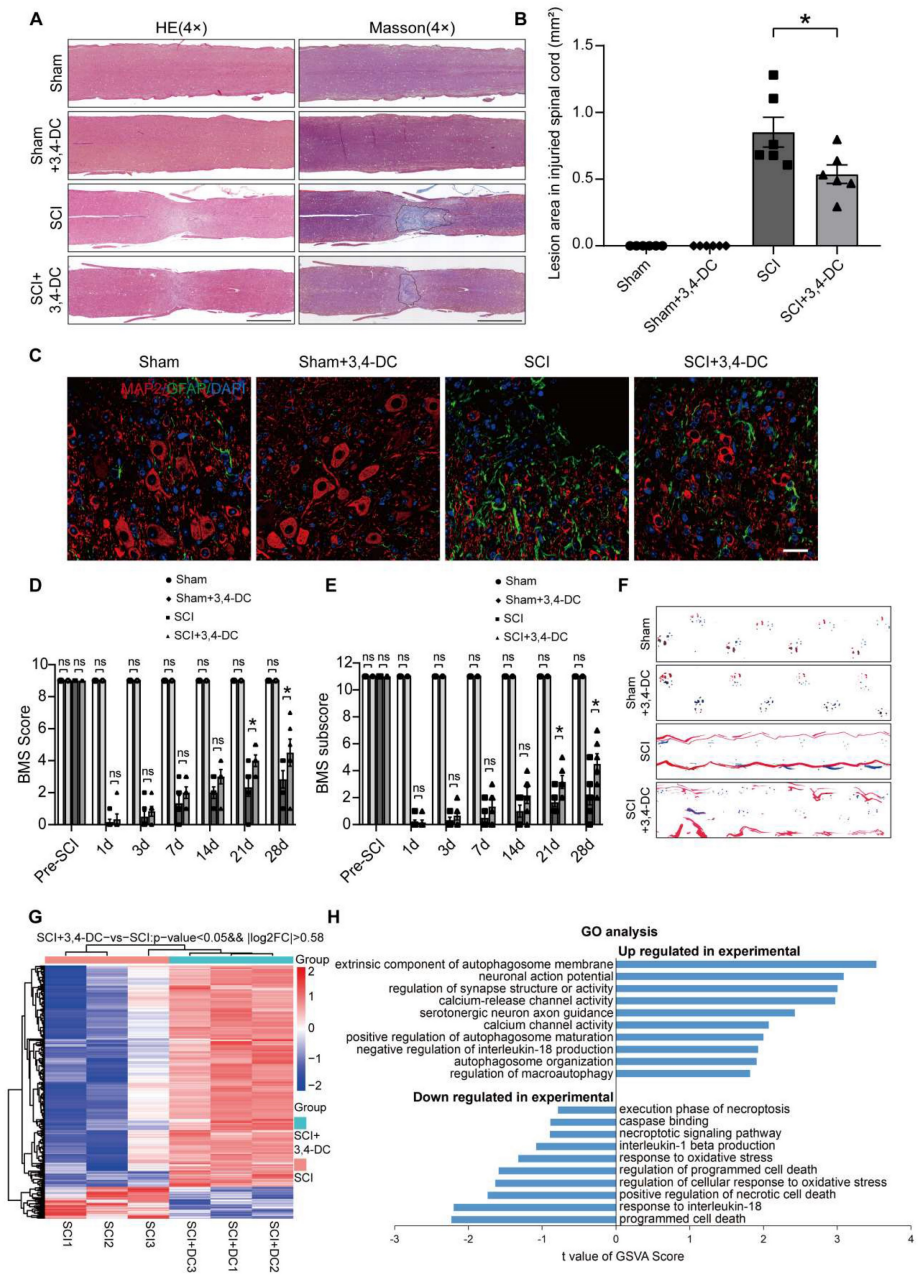
** 3,4-DC improves functional restoration after SCI. (A)** Longitudinal spinal cord sections obtained from the groups on day 28 after SCI were examined by performing HE staining and Masson staining; scale bar: 1,000 μm. **(B)** Quantification of Masson-positive lesions in the spinal cords of all groups. **(C)** Typical images of immunofluorescence staining for MAP2 (red) and GFAP (green) and DAPI staining (blue) in sagittal sections of thoracic spinal cords on day 28 after SCI; scale bar: 100 μm. **(D-E)** BMS score and subscore of the groups on days 1, 3, 7, 14, 21 and 28 after SCI. **(F)** Representative images of footprints used in walking analyses of mice on day 28 after SCI. Blue: forepaw print; Red: hindpaw print. **(G)** Heatmap analysis of upregulated and downregulated genes induced by 3,4-DC injection for 3 days into the mouse spinal cord (*n =* 3 mice per group). **(H)** GO enrichment analyses of the targeted genes indicating the bioprocesses affected by 3,4-DC therapy. The data are presented as the means ± SEMs (*n =* 6 mice per group); *P < 0.05, **P < 0.01, and ***P < 0.001 indicate significant differences; ns, not significant. Significance for Fig. 1G and H was calculated using an unpaired t test. Significance for Fig. 1B, D and E was calculated using two-way ANOVA followed by Tukey's multiple comparison test.

We assessed acetyl-α-tubulin levels to detect the ability of 3,4-DC to deacetylate proteins and confirmed that 3,4-DC functions as a CRMs. Western blotting showed that the acetyl-α-tubulin level in the Sham+3,4-DC group decreased compared with that in the Sham group** (***p* < 0.05**)**; similarly, the acetylation level in the SCI+3,4-DC group was also suppressed after SCI **(***p* < 0.001, **Figure S1A-B)**. We monitored the effects of different doses of 3,4-DC on motor function recovery in mice to determine the optimal dose and found that the optimal dose of 3,4-DC was 200 mg/kg [F (1,25) = 79.44, *p* < 0.001**, Figure S1C]**. Next, we performed tissue staining and a motor function evaluation to evaluate the neuroprotective effect of 3,4-DC in mice with spinal cord contusions. HE and Masson staining showed that 3,4-DC had no effect on glial scars in normal mice (*p* > 0.9999), and a large glial scar area was observed at the lesion site after SCI (*p* < 0.0001), while the glial scar area was significantly reduced in the SCI+3,4-DC group (*p* = 0.0132, **Figure 1A-B**). Moreover, in the SCI group, the number of anterior horn motor neurons in the spinal cord was significantly reduced on day 14 after SCI (*p* < 0.0001), and the quantity of SYN-positive synapses on the motor neurons was significantly reduced at day 28 after SCI (*p* < 0.0001). However, these parameters were substantially improved in the SCI+3,4-DC group (*p* = 0.0027; *p* = 0.0003), and compared with the Sham group, there was no significant change in the Sham+3,4-DC group (*p* = 0.9998 for **Figure S1D-E**, *p* = 0.9880 for **Figure S1F-G)**. GFAP expression is involved in glial scar formation after SCI, while MAP2 expression is associated with axonal regeneration and repair. Confocal analysis showed that MAP2 was sparsely distributed at the lesion site in the SCI group compared with the Sham group and Sham+3,4-DC group (*p* < 0.0001; *p* < 0.0001), whereas the MAP2 distribution density was significantly increased after 3,4-DC treatment (*p* = 0.0005). At the same time, GFAP expression was increased in the damaged area of the spinal cord (*p* < 0.0001), and a certain degree of decrease was observed after 3,4-DC treatment (*p* = 0.0009; **Figure 1C**). We performed a footprint analysis and determined BMS score and subscore to further investigate the contribution of 3,4-DC to locomotive functional recovery. Compared with the SCI group, significantly higher BMS score were recorded for 3,4-DC-treated SCI mice on days 21 and 28 (**Figure 1D-E**). We detected a significant effect of 3,4-DC [F_(1,20)_ = 95.22, *p* < 0.001 for BMS score and F_(1,20)_ = 64.59, *p* < 0.001 for BMS subscore]. The footprint analysis performed 28 days after injury showed gait recovery based on hind limb function in the SCI+3,4-DC group, while the SCI group remained unable to raise the hind limbs (**Figure 1F**).

Transcriptome sequencing was performed in the SCI group and 3,4-DC group to investigate the mechanism underlying the effect of 3,4-DC. A total of 318 genes were differentially expressed (278 were upregulated, and 40 were downregulated) between the SCI+3,4-DC group and the SCI group (*p* < 0.05, **Figure 1G**). In addition, GO analysis showed that the DEGs were predominantly enriched in autophagy, oxidative stress, necroptosis, and the IL-1β and IL-18 pathways (**Figure 1H**). The transcriptome sequencing results showed that 3,4-DC may promote autophagy and suppress pyroptosis and necroptosis to promote functional recovery after SCI.

### 3,4-DC attenuates pyroptosis after SCI

**Figure 2:**
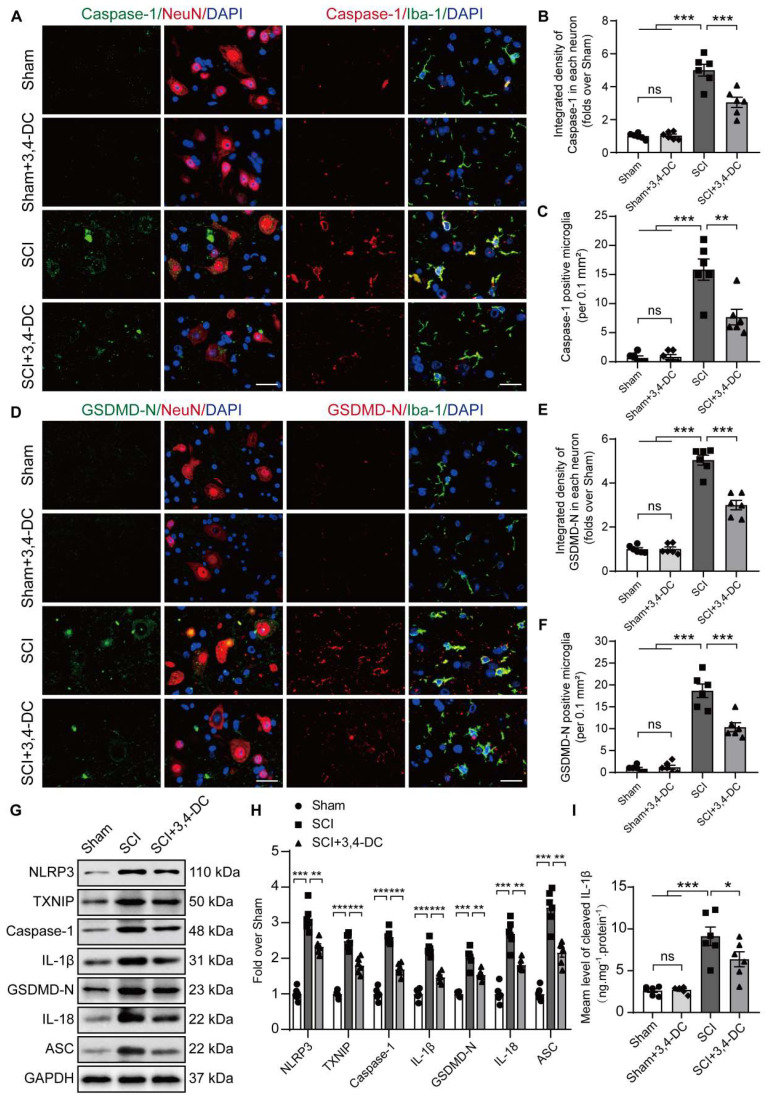
** 3,4-DC attenuates pyroptosis after SCI. (A)** Typical immunofluorescence staining images of the spinal cord ventral horn grey matter showing caspase-1 and NeuN colocalization and caspase-1 and Iba-1 colocalization in the indicated groups on day 3 after SCI; scale bar: 20 μm. **(B-C)** Graph showing the relative intensities of caspase-1 immunofluorescence staining in neurons and the number of caspase-1-positive microglia in the indicated groups. **(D)** Typical immunofluorescence staining images of spinal cord ventral horn grey matter showing GSDMD-N and NeuN colocalization and GSDMD-N and Iba-1 colocalization in the indicated groups on day 3 after SCI; scale bar: 20 μm. **(E-F)** Graph showing the relative intensities of GSDMD-N immunofluorescence staining in neurons and the number of GSDMD-N-positive microglia in the indicated groups. **(G-H)** WB analysis and quantification of NLRP3, TXNIP, caspase-1, IL-1β, GSDMD-N, IL-18 and ASC protein levels in spinal cord lesions from the indicated groups on day 3 after SCI. GAPDH was utilized as a loading control. **(I)** 3,4-DC treatment attenuated the activation of IL-1β in spinal cord lesions, as detected using ELISA kits. The data are presented as the means ± SEMs (*n =* 6 mice per group); *P < 0.05, **P < 0.01, and ***P < 0.001 indicate significant differences; ns, not significant. Significance was calculated using two-way ANOVA followed by Tukey's multiple comparison test.

In recent years, a large amount of literature has supported TXNIP as a key target for regulating pyroptosis 44. The present study evaluated the expression of pyroptosis-associated molecules to determine whether 3,4-DC suppresses pyroptosis. The levels of critical pyroptosis-associated proteins, including NLRP3, caspase-1, GSDMD-N, IL-1β, IL-18, and ASC, were assessed to evaluate pyroptotic activity. Through immunofluorescence staining, the fluorescence intensities of caspase-1 and GSDMD-N in neuronal cells were detected after SCI (**Figure 2A-C**). The numbers of caspase-1- and GSDMD-N-positive microglial cells were detected after SCI (**Figure 2D-F**). We found that 3,4-DC had no significant effect on pyroptosis levels in normal mouse neurons and microglial cells (*p* = 0.9997 for **Figure 2B**, *p* > 0.9999 for **Figure 2C**, *p* = 0.9943 for **Figure 2E**, *p* > 0.9999 for **Figure 2F**). Caspase-1 and GSDMD-N expression levels were markedly reduced after 3,4-DC treatment compared to levels in the SCI group (*p* = 0.0003 for **Figure 2B**; *p* = 0.001 for **Figure 2C**; *p* < 0.0001 for **Figure 2E**; *p* < 0.0001 for **Figure 2F**). We first analysed the expression of TXNIP and pyroptosis-related proteins in Sham group and Sham+3,4-DC group via WB **(Figure S2A)**. The results showed that 3,4-DC had no significant effect on the level of pyroptosis in normal mice **(***p* = 0.8763, *p* = 0.7803, *p* > 0.9999, *p* = 0.9710, *p* = 0.9836, *p* = 0.7072, *p* = 0.8521, **Figure S2B)**. Then, WB was utilized to analyse the expression of TXNIP and pyroptosis-related proteins in the Sham, SCI, and SCI+3,4-DC groups (**Figure 2G**). TXNIP and pyroptosis-related protein expression increased significantly after SCI compared to that in the Sham group (*p* < 0.0001, *p* < 0.0001, *p* < 0.0001, *p* < 0.0001, *p* = 0.0003, *p* < 0.0001, *p* < 0.0001). However, the expression of all 7 proteins decreased after 3,4-DC treatment (*p* = 0.0042, *p* = 0.0007,* p* < 0.0001, *p* < 0.0001, *p* = 0.0093, *p* = 0.0031, *p* = 0.0015, **Figure 2H**). In addition, we used ELISA kits to detect the levels of the cleaved forms of IL-1β; the activity was significantly decreased after 3,4-DC treatment **(***p* = 0.0325, **Figure 2I)**. These results indicate that 3,4-DC effectively reduces pyroptosis after SCI.

### 3,4-DC inhibits necroptosis after SCI

**Figure 3:**
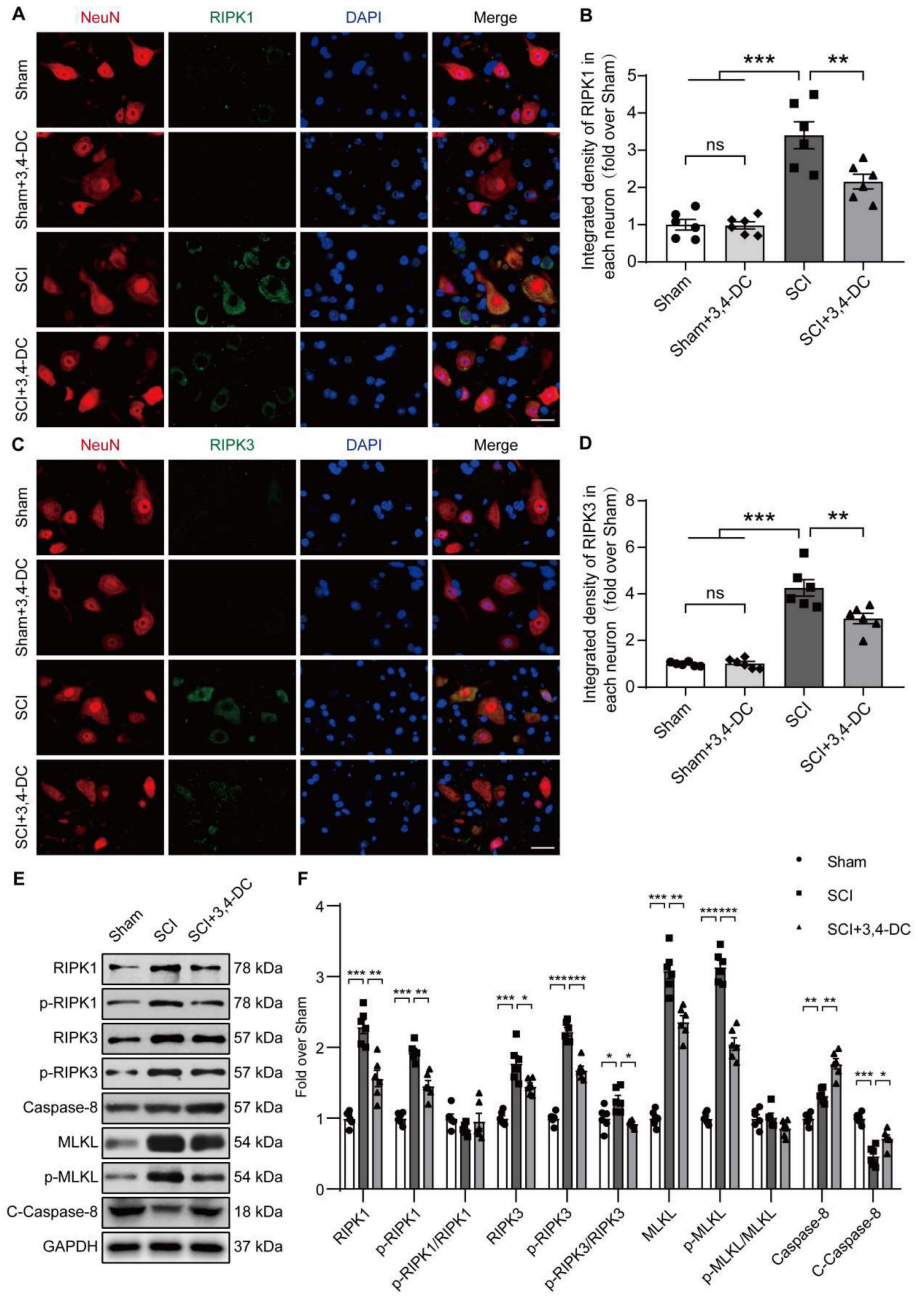
** 3,4-DC inhibits necroptosis after SCI. (A-B)** Double immunofluorescence staining for RIPK1 and NeuN in spinal cord ventral horn grey matter from the groups on day 3 after SCI; scale bar: 20 μm. Quantification of RIPK1 immunofluorescence staining is presented on the right of the representative images. **(C-D)** Double immunofluorescence staining for RIPK3 and NeuN in the spinal cord ventral horn grey matter from the groups on day 3 after SCI; scale bar: 20 μm. Quantification of RIPK3 immunofluorescence staining is presented on the right of the representative images. **(E-F)** WB analyses of necroptosis-associated biomarkers (RIPK1, p-RIPK1, RIPK3, p-RIPK3, MLKL, p-MLKL, caspase-8, and cleaved caspase-8) in spinal cord lesions on day 3 after SCI. Densitometry quantifications are presented on the right. GAPDH was utilized as a loading control. The data are presented as the means ± SEMs (*n =* 6 animals per group); *P < 0.05, **P < 0.01, and ***P < 0.001 indicate significant differences; ns, not significant. Significance was calculated using two-way ANOVA followed by Tukey's multiple comparison test.

Based on accumulating evidence, strategies targeting necroptosis may help to inhibit multiple cell death pathways and attenuate neuroinflammation 45. Therefore, we performed immunofluorescence staining and WB to detect necroptosis activity in spinal cord tissue. The double-staining data showed that the fluorescence densities of RIPK1 and RIPK3 in neurons decreased significantly after treatment of SCI with 3,4-DC (*p* = 0.0091, **Figure 3A-B** and *p* = 0.0083, **Figure 3C-D**). In addition, the RIPK1, p-RIPK1, RIPK3, p-RIPK3, MLKL, p-MLKL, caspase-8, and cleaved caspase-8 levels between the Sham and Sham+3,4-DC groups were analysed using WB (**Figure S2C**). The results showed that 3,4-DC had no effect on the level of necroptosis in normal mice (*p* = 0.6028, *p* = 0.3831, *p* = 0.8983, *p* = 0.9577, *p* = 0.5423, *p* = 0.5368, *p* = 0.9159, *p* = 0.7258, **Figure S2D**). Compared to the Sham group, the RIPK1, p-RIPK1, RIPK3, p-RIPK3, MLKL and p-MLKL levels were markedly increased in the SCI group (*p* < 0.0001, *p* < 0.0001, *p* < 0.0001, *p* < 0.0001, *p* < 0.0001, *p* < 0.0001), indicating the activation of necroptosis after SCI **(Figure 3E)**. This finding was consistent with previously obtained RNA sequencing data. Compared to the SCI group, the RIPK1, p-RIPK1, RIPK3, p-RIPK3, MLKL, and p-MLKL protein levels decreased significantly after 3,4-DC therapy, while the activity of cleaved caspase-8 increased significantly (*p* = 0.0018, *p* = 0.0012, *p* = 0.0431, *p* = 0.0001, *p* = 0.002, *p* < 0.0001, *p* = 0.0017, *p* = 0.0197, **Figure 3F**). Collectively, these outcomes suggest that the protective effect of 3,4-DC in SCI is partially due to the inhibition of necroptosis.

### 3,4-DC reinforces autophagy activity after SCI

**Figure 4:**
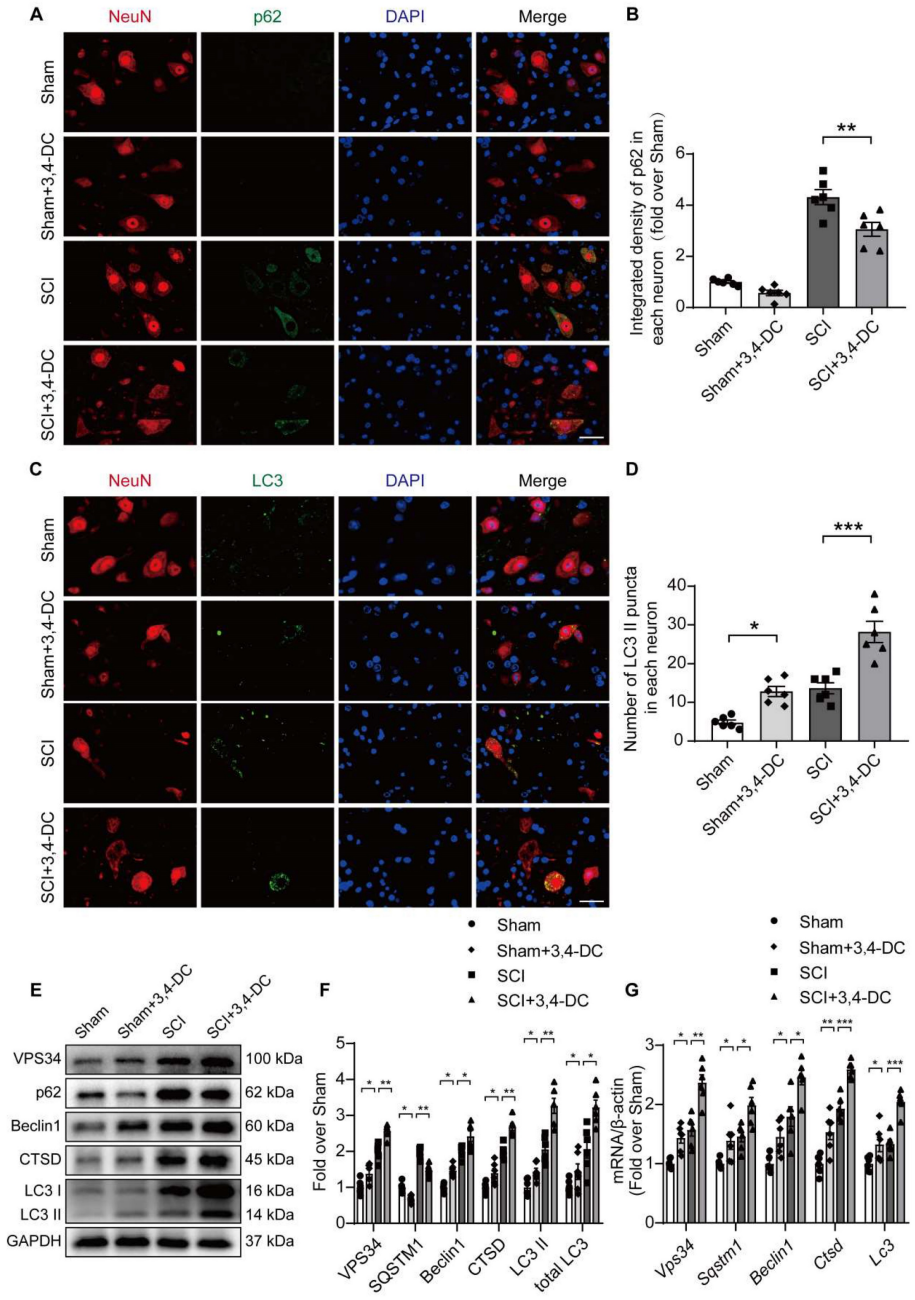
** 3,4-DC reinforces autophagy activity after SCI. (A-B)** Double immunofluorescence staining showing p62 and NeuN colocalization in spinal cord ventral horn grey matter from all groups (Sham, Sham+3,4-DC, SCI and SCI+3,4-DC groups) on day 3 after SCI; scale bar: 20 μm. Quantification of p62 immunofluorescence staining is presented on the right of the representative images. **(C-D)** Double immunofluorescence staining showing LC3 and NeuN colocalization in the spinal cord ventral horn grey matter from all groups (Sham, Sham+3,4-DC, SCI and SCI+3,4-DC groups) on day 3 after SCI; scale bar: 20 μm. The number of LC3II puncta in each neuron is presented on the right. **(E-F)** WB analyses of VPS34, p62, Beclin1, CTSD and LC3 in the injured spinal cord lesion areas on day 3 after SCI. GAPDH was utilized as a loading control. The illustrations on the right present the summarized data from WB analyses. **(G)** Relative mRNA levels of autophagy-related genes in the injured spinal cord of the groups on day 3 after SCI. The data were normalized to *β-actin* and are presented as the means ± SEMs (*n =* 6 animals per group); *P < 0.05, **P < 0.01, and ***P < 0.001 indicate significant differences; ns, not significant. Significance was calculated using two-way ANOVA followed by Tukey's multiple comparison test.

Autophagy has been used as a target to treat CNS illnesses and is involved in the regulation of programmed cell death caused by neuroinflammation 27. We examined the expression of autophagy-associated proteins, including autophagosome markers (VPS34, Beclin1, and LC3), the lysosomal enzyme cathepsin D (CTSD), and an autophagic substrate protein (p62) 46, to assess the effects of autophagy activity on SCI after treatment with 3,4-DC. As presented in **Figure 4A-B**, the expression of p62 in neurons in the spinal cord lesion decreased after 3,4-DC treatment compared with expression in the SCI group (*p* = 0.001). Additionally, immunofluorescence staining showed that the LC3Ⅱ signals in neurons increased after SCI and that the number of LC3Ⅱ puncta was further increased after 3,4-DC treatment (*p* = 0.0004, **Figure 4C-D**). Interestingly, 3,4-DC also seemed to increase LC3Ⅱ puncta in the Sham+3,4-DC group compared to the Sham group (*p* = 0.0449). Moreover, WB revealed that the VPS34, Beclin1, CTSD, LC3Ⅱ, and total LC3 expression levels increased and that of p62 decreased after 3,4-DC treatment (*p* = 0.0033, *p* = 0.0029, *p* = 0.0287, *p* = 0.0016, *p* = 0.0052, *p* = 0.0231, **Figure 4E-F**). Consistently, qPCR results revealed that the *Vps34*,* Sqstm1, Beclin1*,* Ctsd*, and *Lc3* mRNA levels were markedly increased (*p* = 0.0045, *p* = 0.0411, *p* = 0.0439, *p* = 0.0006, *p* = 0.0002, **Figure 4G**). These results confirm that 3,4-DC promotes autophagic lysosomal activity and enhances autophagy in SCI.

### 3,4-DC inhibits necroptosis and pyroptosis by activating autophagy after SCI

**Figure 5:**
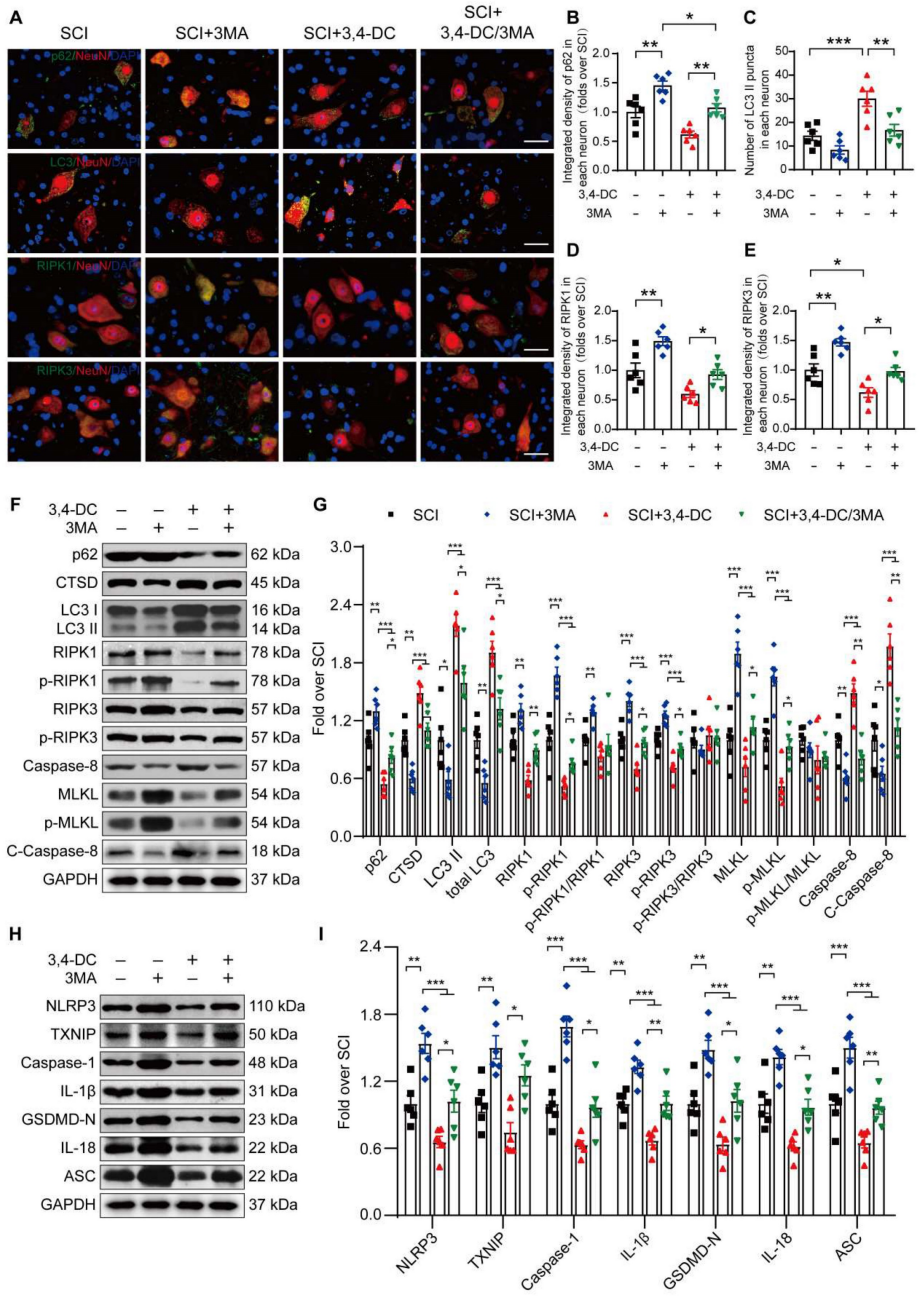
**3,4-DC inhibits necroptosis and pyroptosis by activating autophagy after SCI. (A)** Representative images showing dual immunostaining for p62/NeuN, LC3/NeuN, RIPK1/NeuN and RIPK3/NeuN in the injured spinal cord ventral horn grey matter from each group (the SCI, SCI+3MA, SCI+3,4-DC, and SCI+3,4-DC/3MA groups) on day 3 after SCI; scale bar: 20 μm. **(B-C)** Quantitation of the integrated density of p62 in each neuron and the number of LC3II-positive puncta in neurons are shown in the graph. **(D-E)** The quantitated integrated density of RIPK1 and RIPK3 in spinal cord ventral horn neurons from mice with SCI is shown in the graph. **(F-G)** Typical images of WBs showing CTSD, p62, LC3, RIPK1, p-RIPK1, RIPK3, p-RIPK3, MLKL, p-MLKL, caspase-8 and cleaved caspase-8 levels in the injured spinal cord lesion on day 3 after SCI. **(H-I)** Typical images of WB analyses of TXNIP, NLRP3, caspase-1, IL-1β, GSDMD-N, IL-18, and ASC in the injured spinal cord on day 3 after SCI. GAPDH was utilized as a loading control. The illustrations on the right present the summarized WB data. The data are presented as the means ± SEMs (*n =* 6 mice per group); *P < 0.05, **P < 0.01, and ***P < 0.001 indicate significant differences. Significance was calculated using two-way ANOVA followed by Tukey's multiple comparison test.

We conducted a rescue assay comparing the SCI group, SCI+3MA group, SCI+3,4-DC group, and SCI+3,4-DC/3MA group to further determine the relationship between autophagy, necroptosis and pyroptosis in the SCI model treated with 3,4-DC. Immunofluorescence colocalization analysis revealed that the p62 density in neurons from the SCI+3,4-DC/3MA group was remarkably greater than that in neurons from the SCI+3,4-DC group (*p* = 0.005), and the number of LC3II-positive puncta in neurons was decreased** (***p =* 0.0039, **Figure 5A-C)**. Consistently, in the SCI+3,4-DC/3MA group, WB results revealed that the CTSD, LC3II and total LC3 levels were reduced and the p62 level was increased compared with those levels in the SCI+3,4-DC group **(***p* = 0.0275, *p* = 0.0285,* p* = 0.0386, *p* = 0.0202,** Figure 5F-G)**. Therefore, 3MA inhibits the neuronal autophagy-lysosomal pathway activated by 3,4-DC. The effects of 3MA on necroptosis and pyroptosis were also examined. Immunofluorescence staining revealed that the density of RIPK1 and RIPK3 in neurons from the SCI+3,4-DC/3MA group increased significantly compared to the density in SCI+3,4-DC group **(***p* = 0.0489,** Figure 5D and** *p* = 0.0391,** Figure 5E)**. WB results showed increased expression of necroptosis-related proteins in the SCI+3,4-DC/3MA group **(***p* = 0.0097, *p* = 0.0153,* p* = 0.0273, *p* = 0.0373, *p* = 0.0433, *p* = 0.0143, *p* = 0.0011, *p* = 0.0023, **Figure 5F-G)**. Next, fluorescence colocalization indicated that the fluorescence densities of caspase-1 and GSDMD-N in neuronal cells and the number of caspase-1- and GSDMD-N-positive microglial cells were increased in the SCI+3,4-DC/3MA group **(***p* = 0.0266 for caspase-1, and *p* = 0.0202 for GSDMD-N, **Figure S3A-F)**. Similarly, WB showed increased expression of pyroptosis-related proteins in the SCI+3,4-DC/3MA group **(***p* = 0.0461, *p* = 0.0115, *p* = 0.0492, *p* = 0.0095, *p* = 0.0440, *p* = 0.0118, *p* = 0.0096,** Figure 5H-I)**. Based on these results, 3MA partially reverses the inhibitory effects of 3,4-DC on necroptosis and pyroptosis. Moreover, compared with the SCI, SCI+3MA, and SCI+3,4-DC/3MA groups, 3MA significantly decreased autophagy and promoted necroptosis and pyroptosis in mice suffering from SCI, and additional 3,4-DC treatment reversed these changes. These results reveal that 3,4-DC inhibits necroptosis and pyroptosis by activating autophagy after SCI.

### 3,4-DC promotes functional recovery by enhancing autophagy after SCI

We performed HE, Masson and Nissl staining to observe the histomorphology of the impaired spinal cord tissues from all groups. The glial scar area in injured spinal cord tissue from the SCI+3,4-DC/3MA group was remarkably larger than that in the SCI+3,4-DC group **(***p* = 0.0051, **Figure S4A-B)**, and the number of ventral motor neurons was also significantly reduced **(***p* = 0.0002, **Figure S5A, C)**. Double-immunofluorescence staining for SYN and NeuN showed that when 3,4-DC and 3MA were administered in combination, a few SYN-positive synapses were present on neurons in the injured spinal cord segment **(***p* = 0.0002, **Figure S5B, D)**. In addition, confocal microscopy examination of the SCI site showed that MAP2 expression significantly decreased (*p* = 0.0012) and the GFAP signal significantly increased (*p* = 0.0212) after the SCI+3,4-DC/3MA combined treatment compared with the SCI+3,4-DC group **(Figure S4C-E)**. The BMS score and subscore in the SCI+3,4-DC/3MA group were lower than those in the SCI+3,4-DC group on the 28th day** (Figure S4F-G)**. We detected a significant effect of 3MA [F(1,20) = 67.71, *p* < 0.001 for BMS score and F(1,20) = 158.75, *p* < 0.001 for BMS subscore]. On the 28th day after injury, footprint analysis showed that mice in the SCI+3,4-DC/3MA group were still unable to lift their hind legs. However, the motor function of mice in the SCI+3,4-DC group was improved **(Figure S4H)**. Therefore, the improvement in functional recovery after SCI induced by 3,4-DC may be attributed to an increase in autophagic flux. Moreover, compared with the SCI, SCI+3MA, and SCI+3,4-DC/3MA groups, 3MA treatment significantly inhibited histological and functional recovery in mice suffering from SCI, and additional 3,4-DC treatment reversed these changes. Thus, 3,4-DC promotes functional recovery by enhancing autophagy after SCI.

### 3,4-DC promotes autophagy and subsequently inhibits pyroptosis and necroptosis by upregulating TFEB nuclear translocation after SCI

**Figure 6:**
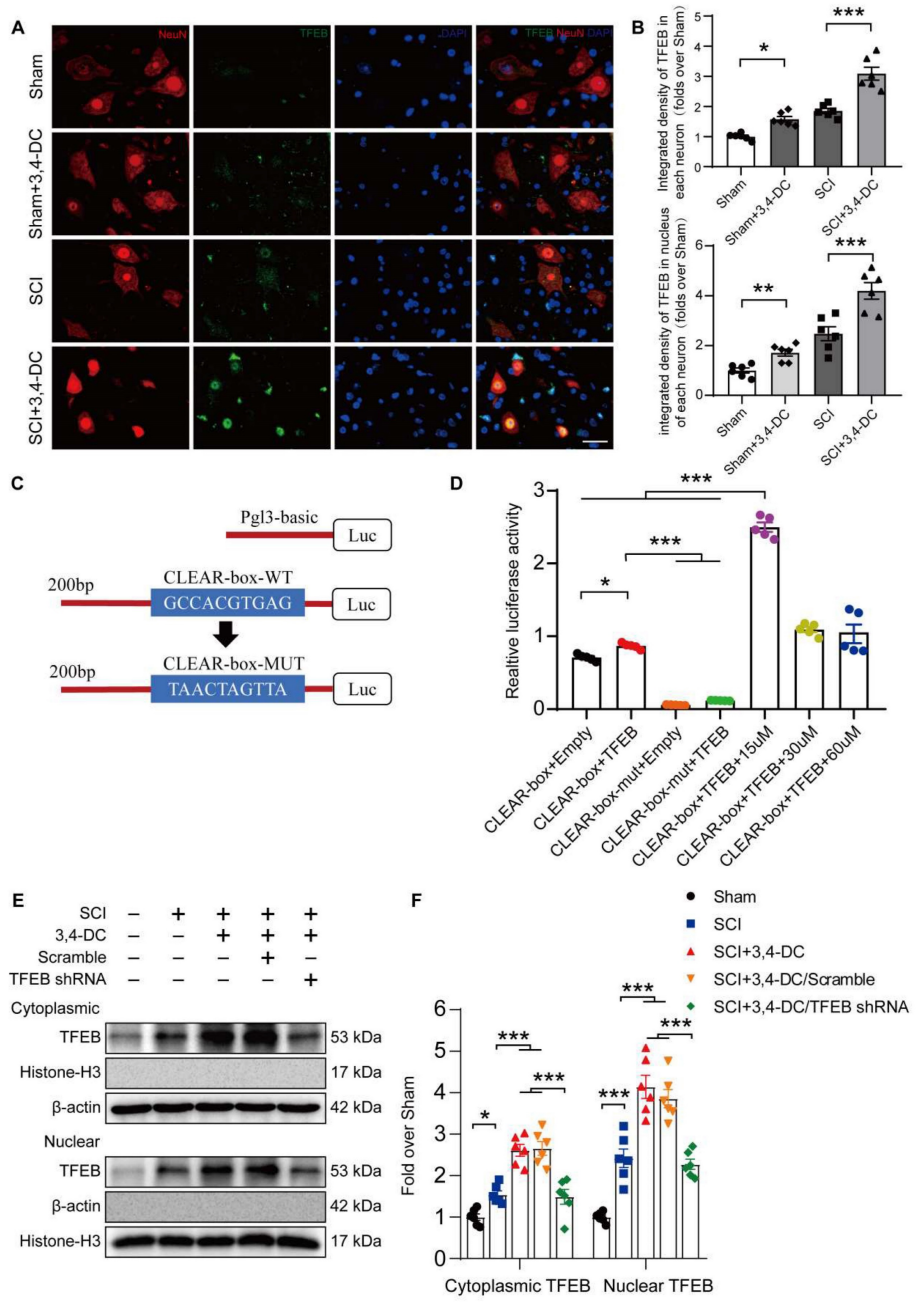
** 3,4-DC enhances TFEB nuclear translocation. (A-B)** Representative images of immunofluorescence staining for TFEB (green) and NeuN (red) in the injured spinal cord ventral horn grey matter on day 3 after SCI. The illustration showing the quantitative results indicates that 3,4-DC increased the integrated density of TFEB in spinal cord neurons and the integrated density of TFEB in the nucleus of each neuron; scale bar: 20 μm. **(C-D)** Mutant CTSD promoter-driven luciferase reporters with mutated bases were constructed, and the relative fluorescence intensity was measured. **(E-F)** WB analysis of cytoplasmic and nuclear TFEB levels in the injured spinal cord lesions on day 3 after SCI in each group (Sham, SCI, SCI+3,4-DC, SCI+3,4-DC/scrambled shRNA, and SCI+3,4-DC/TFEB shRNA groups). The data were normalized to β-actin or histone H3. The data are presented as the means ± SEMs (*n =* 6 mice per group, except for the luciferase reporter assays, where *n =* 5); *P < 0.05, **P < 0.01, and ***P < 0.001 indicate significant differences. Significance was calculated using two-way ANOVA followed by Tukey's multiple comparison test.

Based on accumulating evidence, stimulation of TFEB is a major modulator of autophagy activity and lysosome biogenesis, decreasing neural toxicity and reducing neuroinflammation in animal models 28, 47. Therefore, we detected TFEB levels in the cytoplasm and nucleus to assess whether 3,4-DC regulates autophagy through TFEB in the SCI model. As presented in **Figure 6A-B**, TFEB expression in neurons increased significantly after 3,4-DC treatment (*p* < 0.001), indicating that 3,4-DC effectively enhanced TFEB nuclear translocation (*p* < 0.001). We further analysed the mechanism by which 3,4-DC increases TFEB expression levels by identifying potential binding sites in the CTSD promoter region of TFEB using the UniProt database (https://www.uniprot.org) (**Figure 6C**). A dual-luciferase reporter assay showed that the relative luciferase activity in the CLEAR-box+TFEB group was increased compared with that in the CLEAR-box+Empty group (*p* < 0.05), indicating that TFEB exhibited a transcriptional function. After the CLEAR-box was mutated, the luciferase activity in the CLEAR-box-mut+TFEB group was significantly reduced compared with that in the CLEAR-box+Empty group (*p* < 0.0001), indicating that the CLEAR-box plays an important role in the transcription process mediated by TFEB. Then, we added 15, 30 or 60 μM 3,4-DC and found that the luciferase activity increased significantly after treatment with 15 μM 3,4-DC (*p* < 0.0001), indicating that 3,4-DC substantially enhanced the transcriptional activity of TFEB and CLEAR-box, but the concentration should not be too high (**Figure 6D**). Therefore, we postulate that 3,4-DC enhances autophagy by regulating TFEB expression at the transcriptional level. Next, our team silenced TFEB expression with TFEB shRNA and developed an assay to compare the following 5 groups: Sham, SCI, SCI+3,4-DC, SCI+3,4-DC/scrambled shRNA, and SCI+3,4-DC/TFEB shRNA. The results revealed significantly lower levels of TFEB in the cytoplasm and nucleus of neurons from the SCI+3,4-DC/TFEB shRNA group than in those from the SCI+3,4-DC/scrambled shRNA group (*p* = 0.0002 for cytoplasm, *p* < 0.0001 for nucleus), but a significant difference was not observed between the SCI+3,4-DC and SCI+3,4-DC/scrambled shRNA groups (*p* > 0.9999 for cytoplasm, *p* = 0.9364 for nucleus, **Figure 6E-F**). Thus, transfection with TFEB shRNA effectively inhibited TFEB expression and nuclear translocation.

**Figure 7:**
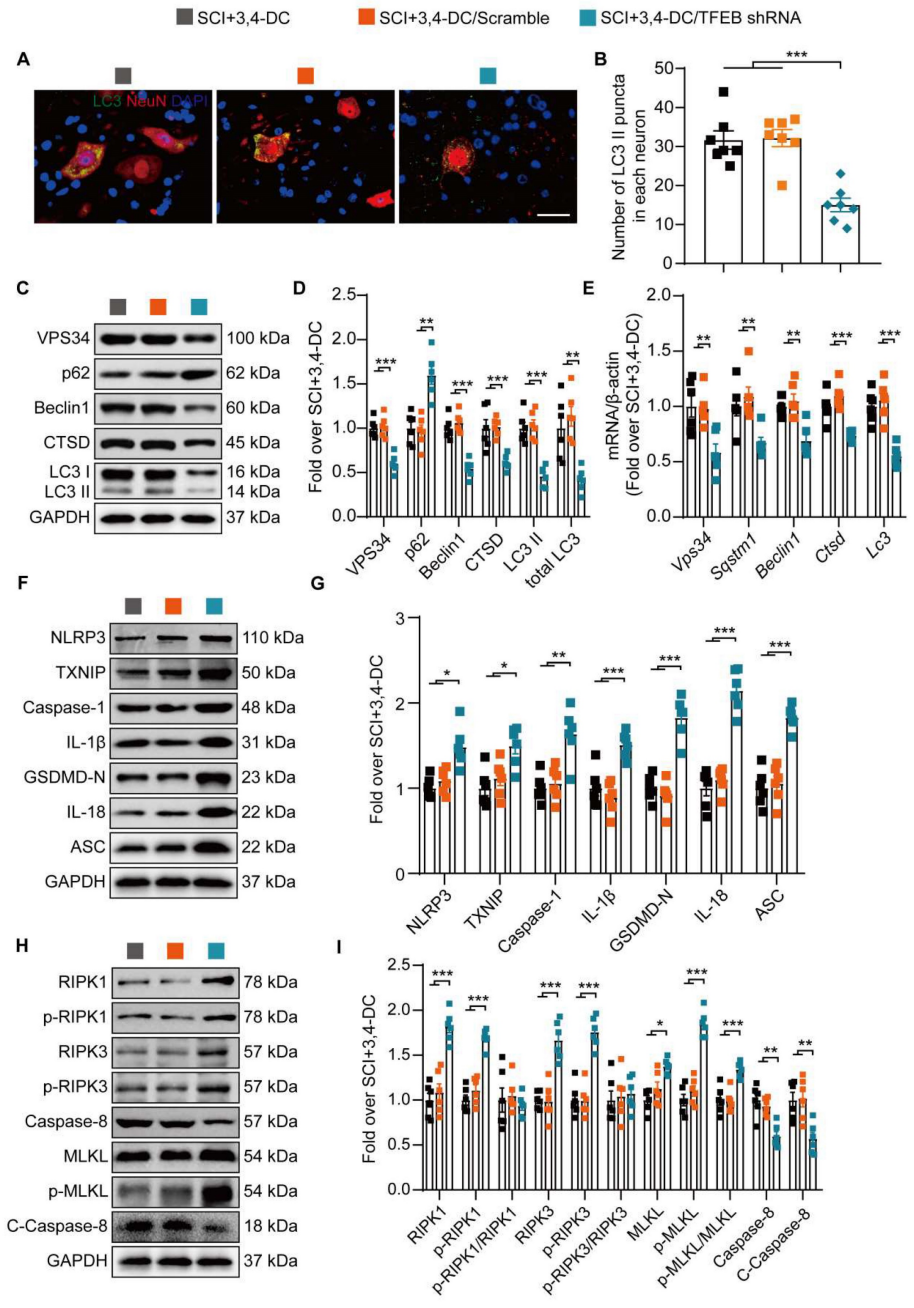
** 3,4-DC promotes autophagy by increasing TFEB expression levels after SCI. (A-B)** Typical immunofluorescence staining images of spinal cord ventral horn grey matter on day 3 after SCI, with the number of LC3II-positive puncta in neurons shown in the graph; scale bar: 20 μm. (C-D) Levels of the VPS34, p62, Beclin1, CTSD, and LC3 proteins in the injured spinal cord tissue on day 3 after SCI. Quantification of the expression of autophagy-associated proteins. GAPDH was utilized as a loading control. **(E)** The expression of autophagy-associated genes in the injured spinal cord on day 3 after SCI was detected using qPCR. The data were normalized to *β-actin*. **(F-I)** WB analysis of TXNIP, NLRP3, caspase-1, IL-1β, GSDMD-N, IL-18, ASC, RIPK1, p-RIPK1, RIPK3, p-RIPK3, MLKL, p-MLKL, caspase-8, and cleaved caspase-8 levels in injured spinal cord lesions on day 3 after SCI. GAPDH was utilized as a loading control. Densitometry quantification of the expression of TXNIP, pyroptosis-, and necroptosis-related proteins in the injured spinal cord lesions. The data are presented as the means ± SEMs (*n =* 6 mice per group); *P < 0.05, **P < 0.01, and ***P < 0.001 indicate significant differences. Significance was calculated using two-way ANOVA followed by Tukey's multiple comparison test.

Next, our team examined whether the 3,4-DC-induced nuclear translocation of TFEB was responsible for regulating the autophagy-lysosomal pathway, pyroptosis, and necroptosis. Immunofluorescence staining did not reveal remarkable differences in the number of LC3II-positive puncta in neurons between the SCI+3,4-DC group and the SCI+3,4-DC/scrambled shRNA group (*p* = 0.9819), while the number of LC3Ⅱ puncta in the SCI+3,4-DC/TFEB shRNA group was markedly decreased (*p* = 0.0001, **Figure 7A-B**). Likewise, WB showed no significant differences in the expression of autophagy-associated proteins (VPS34, p62, Beclin1, CTSD and LC3) between the SCI+3,4-DC and SCI+3,4-DC/scrambled shRNA groups (*p* = 0.9584, *p* = 0.9365, *p* = 0.6466, *p* = 0.9882, *p* = 0.9362); however, in the SCI+3,4-DC/TFEB shRNA group, the expression of VPS34, Beclin1, CTSD, LC3II and total LC3 was markedly decreased, and the expression of p62 was markedly increased (*p* = 0.0002, *p* = 0.0015, *p* < 0.0001, *p* < 0.0001, *p* = 0.0001, *p* = 0.0014, **Figure 7C-D**). Similarly, real-time quantitative PCR assays indicated that the transcription of autophagy-related genes was reduced after transfection with TFEB shRNA (*p* = 0.0072, *p* = 0.0076, *p* = 0.0048, *p* = 0.0006, *p* = 0.0006, **Figure 7E**). In addition, WB results showed that the levels of TXNIP, pyroptosis-related proteins (NLRP3, Caspase-1, IL-1β, GSDMD-N, IL- 18 and ASC) and necroptosis-associated proteins (RIPK1, p-RIPK1, RIPK3, p-RIPK3, MLKL, and p-MLKL) were markedly increased and that caspase-8 and cleaved caspase-8 levels were significantly downregulated in the SCI+3,4-DC/TFEB shRNA group (*p* = 0.0193, *p* = 0.0323, *p* = 0.0092, *p* = 0.0002, *p* < 0.0001, *p* < 0.0001, and *p* < 0.0001 for **Figure 7F-G**; *p* **=** 0.0004, *p* < 0.0001, *p* = 0.0005, *p* < 0.0001, *p* = 0.0431, *p* < 0.0001,* p* = 0.0014, and *p* = 0.0091 for **Figure 7H-I**). In summary, these results indicate that 3,4-DC regulates TFEB expression at the transcriptional level and that inducing increased TFEB nuclear translocation is an important mechanism by which 3,4-DC promotes autophagy and inhibits pyroptosis and necroptosis.

Finally, we evaluated the therapeutic effect of 3,4-DC after transfection with TFEB shRNA. As shown in **Figure S6A-B**, the area of the glial scar in the SCI+3,4-DC/TFEB shRNA group was increased compared to that in the SCI+3,4-DC and SCI+3,4-DC/scrambled shRNA groups (*p* < 0.05). The number of ventral motor neurons (*p* = 0.0108) and SYN-positive synapses (*p* = 0.0452) on the neurons was significantly decreased (**Figure S7A-D**). Confocal microscopy showed decreased MAP2 expression (*p* = 0.0356) and increased GFAP expression (*p* = 0.0001) in the SCI+3,4-DC/TFEB shRNA group (**Figure S6C-E**). At 21 and 28 days after SCI, the BMS score and subscore in the SCI+3,4-DC/TFEB shRNA group were remarkably lower than those in the SCI+3,4-DC group and SCI+3,4-DC/scrambled group (**Figure S6F-G**). We detected a significant effect of TFEB shRNA [F(1,15) = 29.27, *p* < 0.001 for BMS score and F(1,15) = 59.18, *p* < 0.001 for BMS subscore]. The footprint analysis performed at 28 days after injury showed that the animals in the SCI+3,4-DC/TFEB shRNA group continued dragging their hind legs (**Figure S6H**). These outcomes indicate that TFEB stimulation and nuclear translocation are the main mechanisms by which 3,4-DC enhances autophagy to treat SCI.

### 3,4-DC activates TFEB through the AMPK-TRPML1-calcineurin signalling pathway

Several studies have shown that TRPML1 regulates autophagy and mediates the fusion of autophagosomes and lysosomes through calcineurin-induced TFEB activation during nutrient deprivation 40, 48. Thus, 3,4-DC may act through calcium signalling pathways. Our team analysed the expression levels of proteins involved in this pathway to determine whether this pathway was stimulated during 3,4-DC therapy for SCI. The results indicated that 3,4-DC increased the p-AMPK level (*p* = 0.0018) and decreased the p-mTOR level (*p* = 0.0168) in the cytoplasm, but no significant differences in AMPK or mTOR expression were observed between the SCI and SCI+3,4-DC groups **(***p* = 0.5753, *p* = 0.6682; **Figure S8A-B)**. As downstream signalling molecules, the activities of TRPML1 and calcineurin were remarkably increased (*p* = 0.0489,* p* = 0.0292; **Figure S8A-B**). Thus, 3,4-DC may activate the AMPK-TRPML1-calcineurin pathway.

**Figure 8:**
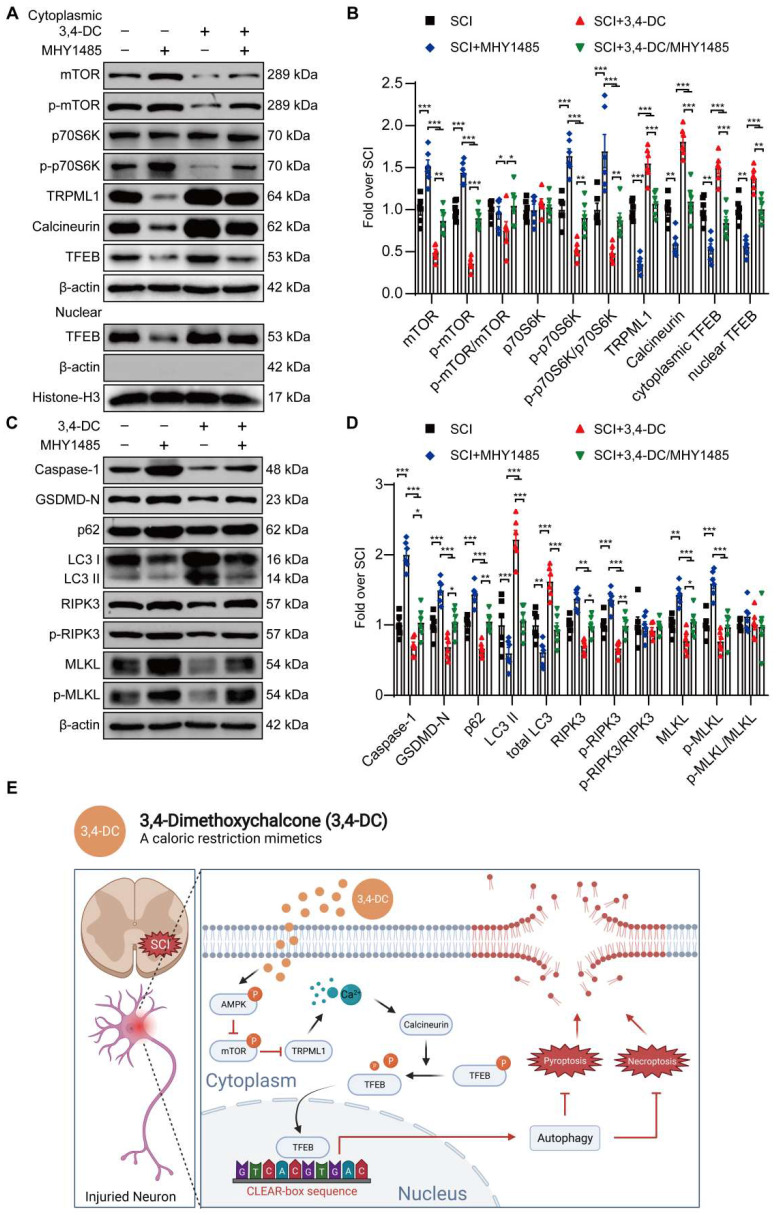
** 3,4-DC activates TFEB through the AMPK-TRPML1-calcineurin signalling pathway. (A-D)** WB analyses showing mTOR, p-mTOR, p70S6K, p-p70S6K, TRPML1, calcineurin, cytoplasmic and nuclear TFEB, caspase-1, GSDMD-N, p62, LC3, RIPK3, p-RIPK3, MLKL, and p-MLKL levels in the injured spinal cord of all groups on day 3 after SCI (the SCI, SCI+MHY1485, SCI+3,4-DC, and SCI+3,4-DC/MHY1485 groups); β-actin or histone H3 was utilized as the loading control. The densitometry quantification shown on the right reveals that MHY1485 (an mTOR agonist) inhibited the 3,4-DC-induced effects. **(E)** Schematic illustrating the proposed molecular mechanism by which 3,4-DC enhances autophagy after SCI by activating the AMPK-TRPML1-calcineurin signalling pathway, thereby promoting the nuclear translocation of TFEB. The induction of autophagy inhibits pyroptosis and necroptosis progression in the injured spinal cord. The data are presented as the means ± SEMs (*n =* 6 mice per group); *P < 0.05, **P < 0.01, and ***P < 0.001 indicate significant differences. Significance was calculated using two-way ANOVA followed by Tukey's multiple comparison test.

Our team explored the effects of Compound C (a known inhibitor of AMPK), MHY1485 (an mTOR agonist) and tacrolimus (a calcineurin suppressor) on the AMPK-TRPML1-calcineurin signalling pathway to determine whether TFEB activation after 3,4-DC therapy was modulated via the AMPK-TRPML1-calcineurin signalling pathway. WB results revealed that 3,4-DC may stimulate the AMPK-TRPML1-calcineurin signalling pathway, but this effect was reversed by Compound C (**Figure S8A-B**), MHY1485 (**Figure 8A-B**) and tacrolimus (**Figure S8C-F).** In addition, MHY1485 significantly inhibited the 3,4-DC-mediated increase in autophagy and inhibited pyroptosis and necroptosis (*p* = 0.0448, *p* = 0.0265, *p* = 0.0013, *p* = 0.0002, *p* = 0.0132, *p* = 0.0068,* p* = 0.0416,* p* = 0.0475, **Figure 8C-D**). In summary, our results confirm that 3,4-DC increases TFEB expression levels in SCI through the AMPK-TRPML1-calcineurin signalling pathway.

## Discussion

Traumatic SCI is a catastrophic and common neurological illness that may cause irreversible nerve damage 49. Current research mainly focuses on the secondary injury stage, with neuroinflammation and programmed cell death identified as the main mechanisms 50. Inflammation is a metabolic reaction requiring intensive metabolic support 51. Existing studies have shown that both intermittent fasting and CR exert anti-inflammatory and neuroprotective effects 52, 53. However, in the absence of malnutrition, deficiencies in patients with strict adherence to intermittent fasting have promoted the emergence of CRMs. The present study firstly investigated the neuroprotective potency of 3,4-DC as a candidate CRMs in a traumatic SCI model. The RNA sequencing analysis results showed that 3,4-DC mainly promoted functional recovery after SCI by regulating autophagy, pyroptosis and necroptosis.

CR is the first known intervention strategy to extend a healthy life and prolong lifespan 53. According to a previous study, CR increases the expression of neuroprotective ketones, antioxidant proteins and antiapoptotic proteins 54. In several nerve injury models, including stroke models, CR has been shown to exert antineuroinflammatory effects and promote functional recovery 55. However, the magnitude and duration of CR should be carefully designed; otherwise, it will interfere with certain physiological functions in the body, limiting its practicality in patients. Therefore, CRMs were designed to simulate the favourable effects of CR without decreasing food consumption. Recently, several drugs and some compounds naturally present in the diet, such as resveratrol, spermidine, and aspirin, have been proven to function as CRMs through various mechanisms. Interestingly, these CRMs exhibit the following three characteristics: (i) the deacetylation of cytoplasmic proteins, (ii) the induction of autophagy, and (iii) a lack of toxicity 53. Similarly, 3,4-DC also has these CRMs characteristics. Previous studies have shown that 3,4-DC protects the heart from ischaemic damage 39.

However, to date, the neuroprotective effect of CRMs on traumatic nerve injury models remains to be elucidated. Therefore, we used 3,4-DC to simulate the effect of CRMs and explore the therapeutic effect of CRMs on SCI. As shown in the present study, the glial scar area of injured spinal cord tissue was significantly reduced after 3,4-DC treatment, and the number of synapses in ventral motor neurons and SYN-positive neurons was significantly increased. The footprint analysis results and BMS score were also significantly improved. According to these observations, we provide the first report that CRMs exert a neuroprotective effect and improve motor function in mice with traumatic SCI. In our study, considering that manually emptying the bladder of male mice with SCI is not easy, we used female mice to establish an SCI model, as described in other studies 56, 57. However, there are sex differences in acute neuroinflammation and neurological recovery in experimental models of SCI 58. On the one hand, oestrogen was found to attenuate SCI by inhibiting proinflammatory pathways, oxidative stress, and cell death 59. Others argue a protective role of testosterone on motoneuron and muscle morphology after SCI 60. Therefore, the impact of sex on SCI is a hot topic. Whether 3,4-DC has different therapeutic effects on SCI under the action of different sex hormones is still unknown. Therefore, in the future, studying the therapeutic effect of 3,4-DC on SCI in male mice would provide valuable information.

Pyroptosis is a recently identified mode of regulated programmed cell death accompanied by inflammation 8. This new type of proinflammatory programmed cell death regulates the activity of NLRP3 inflammasomes by activating caspase-1-, caspase-4/5/11- and GSDMD-modulated signalling pathways, ultimately amplifying the inflammatory response cascade 61. Based on accumulating evidence, TXNIP interacts with NLRP3 inflammasomes in an inflammatory state to activate the TXNIP/NLRP3 pyroptosis pathway 62, 63. Therefore, inhibiting the aforementioned pathway is very important for the treatment of SCI. According to the results of the present study, 3,4-DC significantly reduces the expression of TXNIP and pyroptosis-associated proteins. These results provide the first clarification of the mechanism by which 3,4-DC inhibits pyroptosis after SCI. Autophagy exerts an inhibitory effect on pyroptosis by selectively degrading the NLRP3 inflammasome 64. Here, we found that 3,4-DC treatment promoted neuronal autophagy. Furthermore, inhibition of autophagy by 3MA partially reversed pyroptosis activity and functional recovery after 3,4-DC treatment. In addition, a previous study showed that potassium efflux and the release of cathepsin B (CTSB) are major activators of the NLRP3 inflammasome, and a lack of CTSB markedly reduces NLRP3 activation 65. Autophagy blockade and impaired lysosomal function lead to the release of CTSB 66. However, researchers have not clearly determined whether 3,4-DC maintains autophagy-lysosomal activity in part by inhibiting CTSB leakage to decrease pyroptosis levels in subjects with SCI. Therefore, further research is needed to determine the effect and mechanism of 3,4-DC.

Necroptosis is a form of modulated necrosis that is stimulated downstream of TNFR1; relies on the activity of RIPK1 and RIPK3; is mediated by the mixed-lineage pseudokinase MLKL; and may be caused by mechanical damage, inflammation, or infection 67. Recently, an increasing number of studies have revealed that necroptosis is vital in CNS illnesses and traumatic ischaemic diseases 15, 20. The activation of neuronal necroptosis causes nerve cell death and tissue damage. Hence, our team utilized traditional molecular approaches to determine whether 3,4-DC inhibits necroptosis caused by SCI. This study presents the first data indicating that CRMs significantly inhibit the expression of necroptosis-related proteins, suggesting that CRMs inhibit necroptosis after SCI. Necroptosis often occurs concurrently with autophagy activation, and these processes regulate each other. For example, the autophagy receptor optineurin (OPTN) actively regulates the proteasomal turnover of RIPK1, suggesting that the loss of OPTN induces RIPK1-dependent necroptosis 68. In addition, autophagy-related protein 16-like 1 (ATG16L1) or autophagy-related protein 7 (ATG7) increase autophagic flux and inhibit necroptosis 69. Our results showed that inhibition of autophagy reversed necroptosis activity in neurons to some extent after 3,4-DC treatment of SCI. Therefore, the interaction between autophagy and necroptosis may play a key role in promoting neuronal survival and functional recovery after 3,4-DC treatment of SCI, but the specific mechanism requires further study.

We examined the upstream autophagy mechanism to clarify the mechanism by which 3,4-DC treats SCI. Studies have shown that 3,4-DC triggers the autophagy-lysosomal pathway by activating TFEB 39. As shown in our previous study, TFEB-mediated restoration of autophagy promotes functional recovery in an SCI model 31. Therefore, in this study, we selected TFEB as a possible target for further research. From a mechanistic perspective, under normal physiological conditions, TFEB is usually phosphorylated by mTORC1 kinase at S142 and/or S211 and is retained in the cytoplasm by binding to the cytoplasmic chaperone 14-3-3 protein 48. However, stimulation due to changes in the cellular energy status, such as those mediated by starvation, inhibits mTOR activity, thereby initiating the dephosphorylation of TFEB. Dephosphorylated TFEB is transferred to the nucleus and binds to the CLEAR element, a palindromic 10-base pair motif (*GTCACGTGAC*) in the promotors of genes that regulate autophagy activity and lysosome biogenesis, to stimulate the transcriptional process 70. In this study, a dual-luciferase reporter experiment revealed that 3,4-DC enhances the transcriptional activities of TFEB, thereby regulating lysosomal biogenesis and autophagy. Meanwhile, in the SCI group, TFEB activation markedly increased the transcription and expression levels of autophagy-associated genes, reduced pyroptosis, inhibited necroptosis, and promoted functional recovery. In summary, 3,4-DC exerts a therapeutic effect by facilitating TFEB nuclear translocation. A recent study reported that Yes-associated protein (YAP), a transcriptional cofactor and a major terminal effector of the Hippo pathway, is involved in cellular energy metabolism and interacts with TFEB to enhance autophagic flux and lysosome biogenesis 71. However, the role of the YAP/TFEB pathway in SCI remains unclear. Therefore, the function of TFEB combined with YAP in promoting autophagic flux should be investigated in the context of SCI in the future.

Given the satisfactory treatment potency of 3,4-DC, our team hoped to reveal how 3,4-DC modulates TFEB activity. RNA sequencing analysis revealed increased calcium channel activity in the SCI+3,4-DC group. Notably, a previous study identified AMPK as a key responder to starvation and low-energy states, and AMPK has been shown to be a key regulator of MITF family activity 72. mTOR is a conserved serine/threonine kinase related to the protein kinase family associated with phosphoinosine-3-kinase (PI3K) 73. AMPK monitors the energy state of cells at all times, and mTOR integrates various metabolic signals (including nutrients and hormones). When ATP is depleted, AMPK activation leads to the inhibition of mTOR signalling, thereby inhibiting protein synthesis, an important pathway by which AMPK conserves cellular energy in a low-energy state 73. Under starvation and lysosomal stress conditions, the inhibition of mTOR and the release of Ca^2+^ from lysosomes mediated by the TRPML1 channel activate calcineurin. Calcineurin is a eukaryotic Ca^2+^- and calmodulin-dependent serine/threonine protein phosphatase. Activation of calcineurin induces dephosphorylation of TFEB and promotes its nuclear translocation 48. In the present study, 3,4-DC treatment of SCI was shown to activate the AMPK-TRPML1-calcineurin signalling pathway. In addition, we used Compound C, MHY1485 and tacrolimus to suppress the 3,4-DC-mediated activation of these signalling pathways. In conclusion, we provide the first report that CRMs promote TFEB nuclear translocation in an SCI model through the AMPK-TRPML1-calcineurin signalling pathway.

We believe that CRMs are a feasible and effective solution and will become an important treatment for SCI. However, some problems remain to be solved before CRMs are administered to patients with SCI in the clinic. (1) As a candidate CRMs, the toxicological effects and related side effects of 3,4-DC must be fully evaluated before its clinical application. (2) A large number of patients with SCI require long-term care and treatment. Intraperitoneal injection is very inconvenient for patients. Further research is required to determine whether oral CRMs are an effective treatment for SCI. (3) To maximize the effects of CRMs therapy on autophagy, the feasibility of combination therapy to obtain synergistic effects should be considered. For example, combining 3,4-DC with different CRMs or behavioural/nutritional approaches (such as fasting and exercise) might produce beneficial effects.

This study still has some shortcomings that must be further explored. We are concerned about the acute effects of CRMs. The level of autophagy begins to block in the acute phase of SCI and the blockade peaks on the 3rd day 74. Therefore, we chose to apply 3,4-DC treatment daily from before SCI induction to 3 days after SCI. However, the timeframe in which 3,4-DC treatment promotes behavioural recovery in an SCI model must be further investigated to determine the most appropriate application time. Future studies should further extend the treatment time and better clarify the dynamic changes in 3,4-DC-mediated autophagy induction after SCI and the long-term effects of CRMs on functional restoration after SCI. At the same time, we achieved a therapeutic effect of 3,4-DC in SCI via intraperitoneal injection. However, 3,4-DC may also act through other mechanisms, such as glucose metabolism 75, nucleic acid metabolism 76, or through an immune mechanism 77, which may affect functional recovery in SCI, and these mechanisms remain to be discovered. Previous studies have found that some CRMs, such as resveratrol and aspirin, inhibit oxidative stress and reduce ROS levels 78, 79. Activation of the antioxidative stress mechanism inhibits pyroptosis and necroptosis of neurocytes by regulating autophagy 80. Whether 3,4-DC promotes autophagy and inhibits pyroptosis and necroptosis by inhibiting oxidative stress remains to be further studied. In addition, other cell death pathways, such as ferroptosis, parthanatos and NETosis, are closely associated with neurological diseases and disorders 81-83. The contribution of CRMs to these forms of cell death is also an issue worth exploring. In addition, we investigated whether the protective effect of 3,4-DC is related to the beneficial effects of CRMs or merely to its activity as an autophagy (TFEB) inducer. To answer this question, all autophagy pathways must be counteracted either genetically or by administration of inhibitors. We currently propose that 3,4-DC acts by activating TFEB to induce autophagy and may also exert a therapeutic effect by modulating other pathways (such as inhibiting the acetyltransferase EP300). According to a previous study, CR induces mitophagy by activating AMPK and sirtuins, promoting the production of mitophagy-related markers, such as Bcl-2 19-kDa interacting protein 3 (BNIP3) and Parkin, and activating signalling pathways that lead to increased mitochondrial health, DNA repair, and autophagy 84. Mitochondria damaged by SCI release the proapoptotic proteins BAX/BAK and members of the caspase family, which promote NLRP3 deubiquitination and inflammasome activation and subsequently activate pyroptosis. Meanwhile, the proapoptotic protein PUMA exacerbates necroptosis after SCI by exposing mitochondrial DNA to cytoplasmic sensors, further stimulating the formation of necrosomes 50. Therefore, clearing damaged mitochondria is also critical for neuronal survival. The selective degradation of damaged mitochondria via autophagy is called mitophagy, which is critical for maintaining mitochondrial homeostasis. As shown in our previous study, increased autophagy promotes mitophagy, thereby improving the prognosis of mice with SCI 31. Therefore, CRMs may play an important role in inducing mitophagy, which remains to be further studied. Finally, deacetylation of TFEB regulates its transcriptional activity, stimulating the induction of lysosomal biogenesis to increase autophagy-related gene transcription, which alleviates amyloid plaque deposition in individuals with Alzheimer's disease (AD) 85. As CRMs deacetylate cytoplasmic proteins, we will investigate the deacetylation of TFEB by CRMs in subjects with SCI.

## Conclusions

3,4-DC promotes TFEB nuclear translocation through the AMPK-TRPML1-calcineurin signalling pathway, thereby enhancing autophagy in SCI. These events lead to the inhibition of pyroptosis and necroptosis in the injured spinal cord **(Figure 8E)**. Ultimately, the findings indicated that CRMs help to restore function after SCI. The current research provides strong evidence supporting the advantages of CRMs in the treatment of SCI. Therefore, we propose that CRMs have potential clinical application value, but their clinical application must be further evaluated and optimized.

## Abbreviations

CR: Caloric restriction
CRMs: CR mimetics
SCI: Spinal cord injury
CNS: Central nervous system
3,4-DC: 3,4-Dimethoxychalcone
AMPK: AMP-activated protein kinase
mTOR: Mammalian target of rapamycin
TRPML1: Transient receptor potential mucolipin 1
TFEB: Transcription factor EB
RIPK1: Receptor-interacting serine/threonine-protein kinase 1
RIPK3: Receptor-interacting serine/threonine-protein kinase 3
MLKL: Mixed lineage kinase domain-like protein
TXNIP: Thioredoxin-interacting protein
NLRP3: NLR family pyrin domain containing 3
ASC: Apoptosis-associated speck-like protein containing a caspase recruitment segment
IL-1β: Interleukin 1β
IL-18: Interleukin 18
GSDMD: Gasdermin D
GO: Gene ontology
WB: Western blotting
AAV: Adeno-associated virus
3MA: 3-Methyladenine
HE: Haematoxylin-eosin
LC3: Microtubule-associated protein 1 light chain 3
CTSD: Cathepsin D
BMS: Basso Mouse Scale
MAP2: Microtubule-associated protein 2
GFAP: Glial fibrillary acidic protein
