# Supplementary material for: 3,4-Dimethoxychalcone, a caloric restriction mimetic, enhances TFEB-mediated autophagy and alleviates pyroptosis and necroptosis after spinal cord injury
Source: Theranostics. 2023 Jan 1;13(2):810–32. doi: 10.7150/thno.78370 (PMC9830432; doi:10.7150/thno.78370)
Supplement: Supplementary file 1 — Supplementary figures and tables. [file thnov13p0810s1.pdf]

Supplementary Information

for

3,4-Dimethoxychalcone, a caloric restriction mimetic, enhances the TFEB-mediated autophagy and alleviates pyroptosis and necroptosis after spinal cord injury

Haojie Zhang et. al

Figure S1

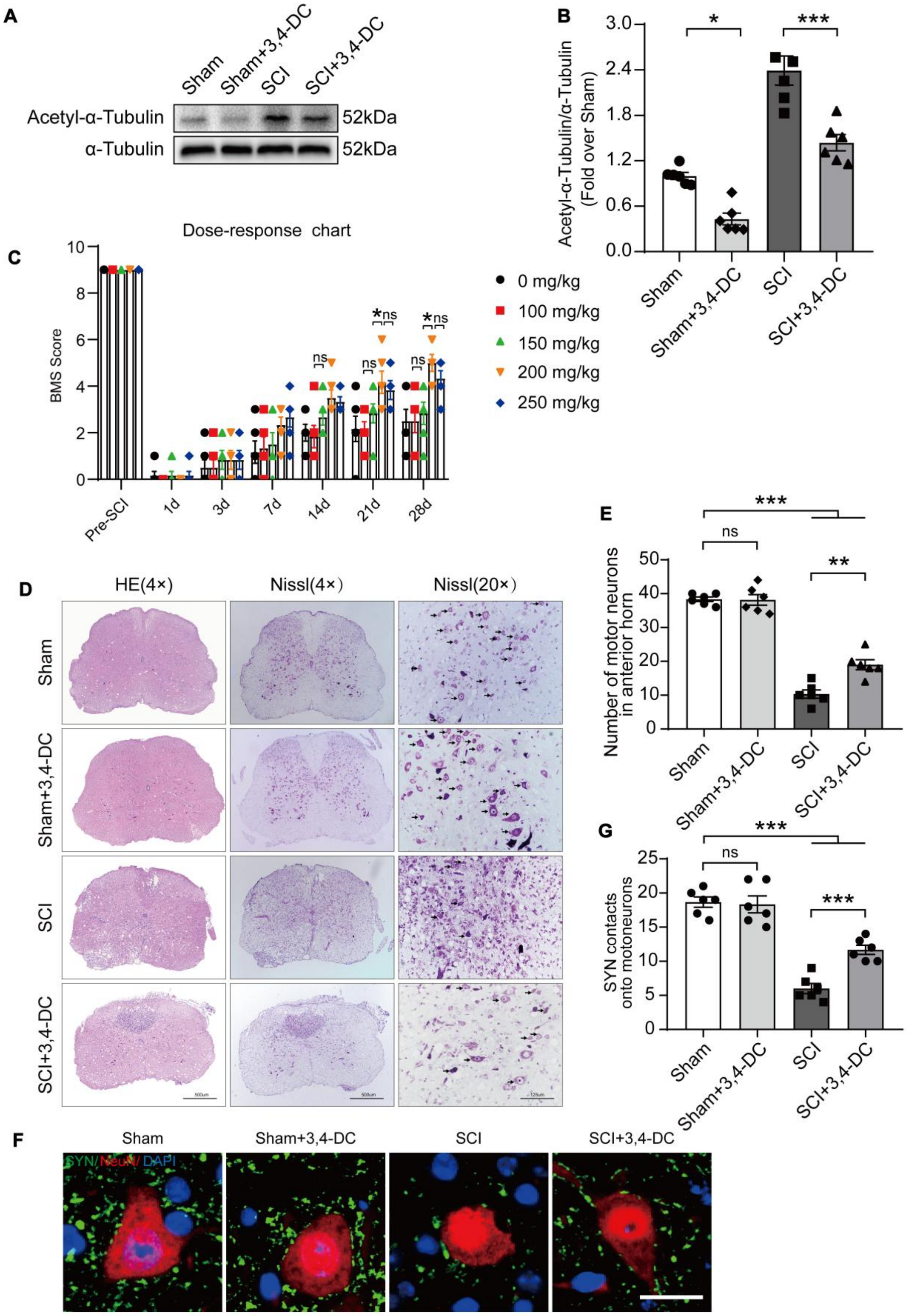

Figure S1. 3,4-DC inhibits motorneuron loss and promotes neuron-synaptic connections after SCI. (A-B) Western blotting analysis and quantification of acetyl-α-tubulin levels in spinal cord lesions in the indicated groups on day 3 after

SCI. α-tubulin was utilized as a loading control. **(C)** Dose–response chart showing the optimal dosage of 3,4-DC (200 mg/kg) for 28 days after SCI for evaluation of BMS score. **(D)** Transverse spinal cord sections from each group were analyzed on day14 by HE staining and Nissl staining; black arrows indicating the motoneurons; scale bar: 500 μm and 125 μm. **(E)** Quantitative analysis of Nissl positive motor neurons in the anterior horn of spinal cord from each group (Sham, Sham+3,4-DC, SCI, and SCI+3,4-DC groups). **(F)** Representative images of spinal cord sections below the injury (T11-T12) and stained on day 28 after SCI with antibodies against SYN/NeuN; scale bar: 5 μm. **(G)** Quantification of the number of synapses contacting onto motor neurons. The data are presented as the means ± SEMs (n = 6 mice per group); *P < 0.05, **P < 0.01, and ***P < 0.001 indicate significant differences; ns, not significant. Significance was calculated using a two-way ANOVA followed by Tukey's multiple comparison test.

Figure S2

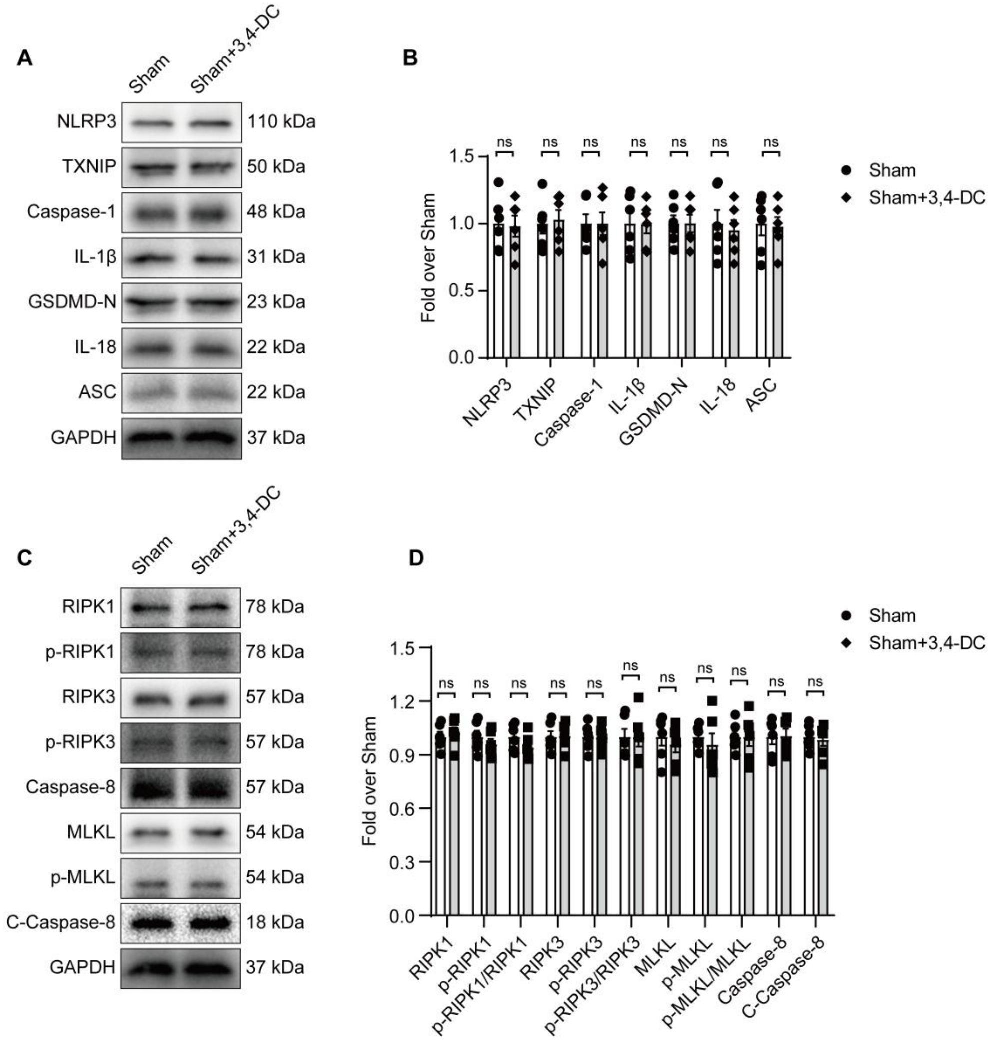

Figure S2. 3,4-DC cannot affect pyroptosis and necroptosis in mice without SCI. (A-B) WB analysis and quantification of NLRP3, TXNIP, caspase-1, IL-1β, GSDMD-N, IL-18 and ASC protein levels in spinal cord lesions from the indicated groups on day 3 after SCI. GAPDH was utilized as a loading control. **(C-D)** WB analyses of necroptosis-associated biomarkers (RIPK1, p-RIPK1, RIPK3, p-RIPK3, MLKL, p-MLKL, caspase-8, and cleaved caspase-8) in spinal cord lesions on day 3 after SCI. Densitometry quantifications are presented on the right. GAPDH was utilized as a loading control. The data are presented as the means ± SEMs (n = 6 mice per group); ns, not significant. Significance was calculated using an unpaired t test.

Figure S3

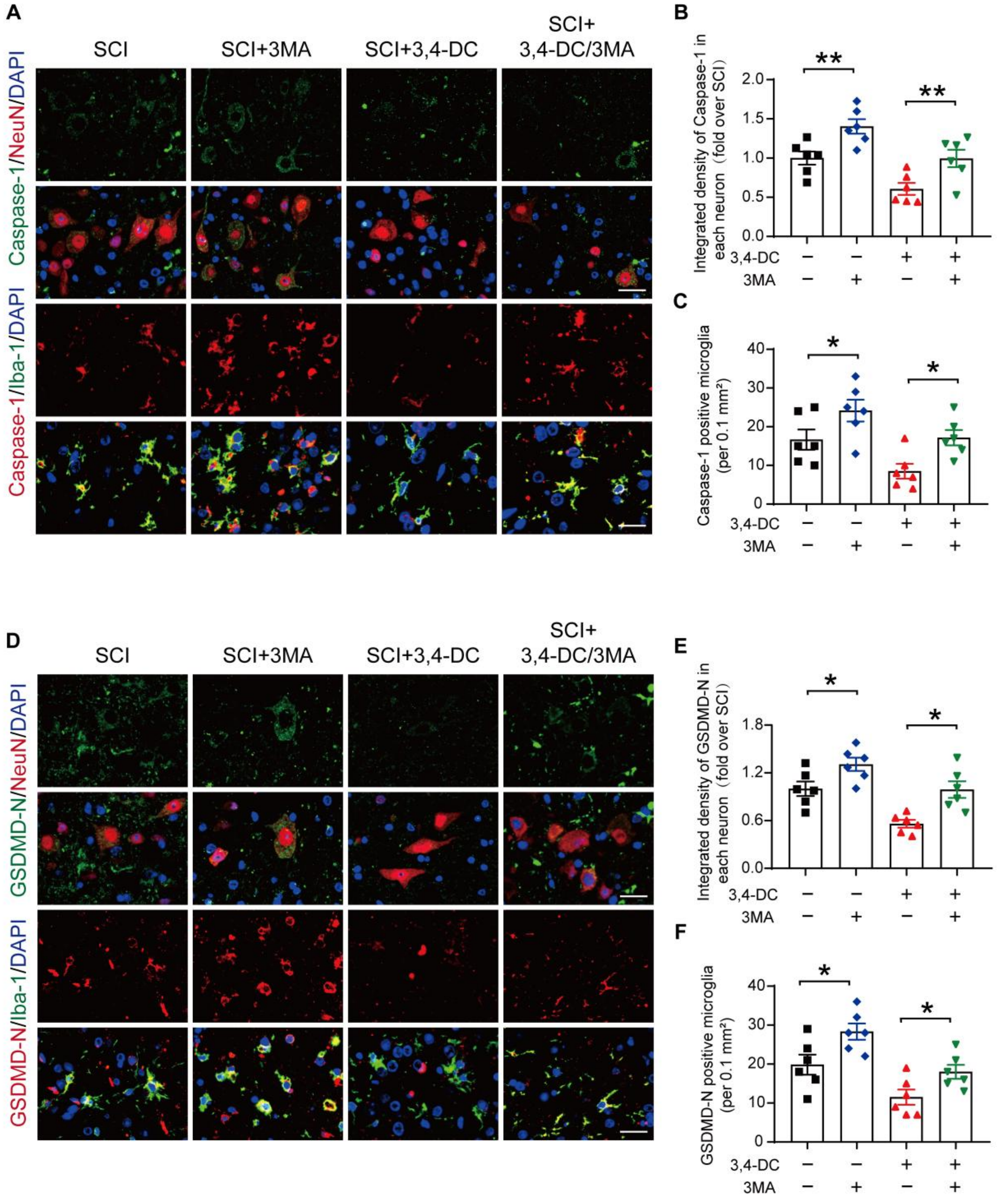

Figure S3. 3,4-DC inhibits pyroptosis by activating autophagy after SCI. (A-C) Representative images of dual immunostaining for caspase-1/NeuN and caspase-1/Iba-1 in spinal cord ventral horns of grey matter from all groups (the SCI,

SCI+3MA, SCI+3,4-DC, and SCI+3,4-DC/3MA groups) on day 3 after SCI; scale bar: 20 μm. The quantitated integrated densities of caspase-1 in each neuron and the number of caspase-1-positive microglia are shown in the graph. **(D-F)** Typical images of dual immunofluorescence staining for GSDMD-N/NeuN and GSDMD-N/Iba-1 on the injured spinal cord on day 3 after SCI; scale bar: 20 μm. The quantitated integrated densities of GSDMD-N in each neuron and the number of GSDMD-N positive microglia are shown in the graph. The data are presented as the means ± SEMs (n = 6 mice per group); *$P < 0.05$, **$P < 0.01$, and ***$P < 0.001$ indicate significant differences; ns, not significant. Significance was calculated using a two-way ANOVA followed by Tukey's multiple comparison test.

Figure S4

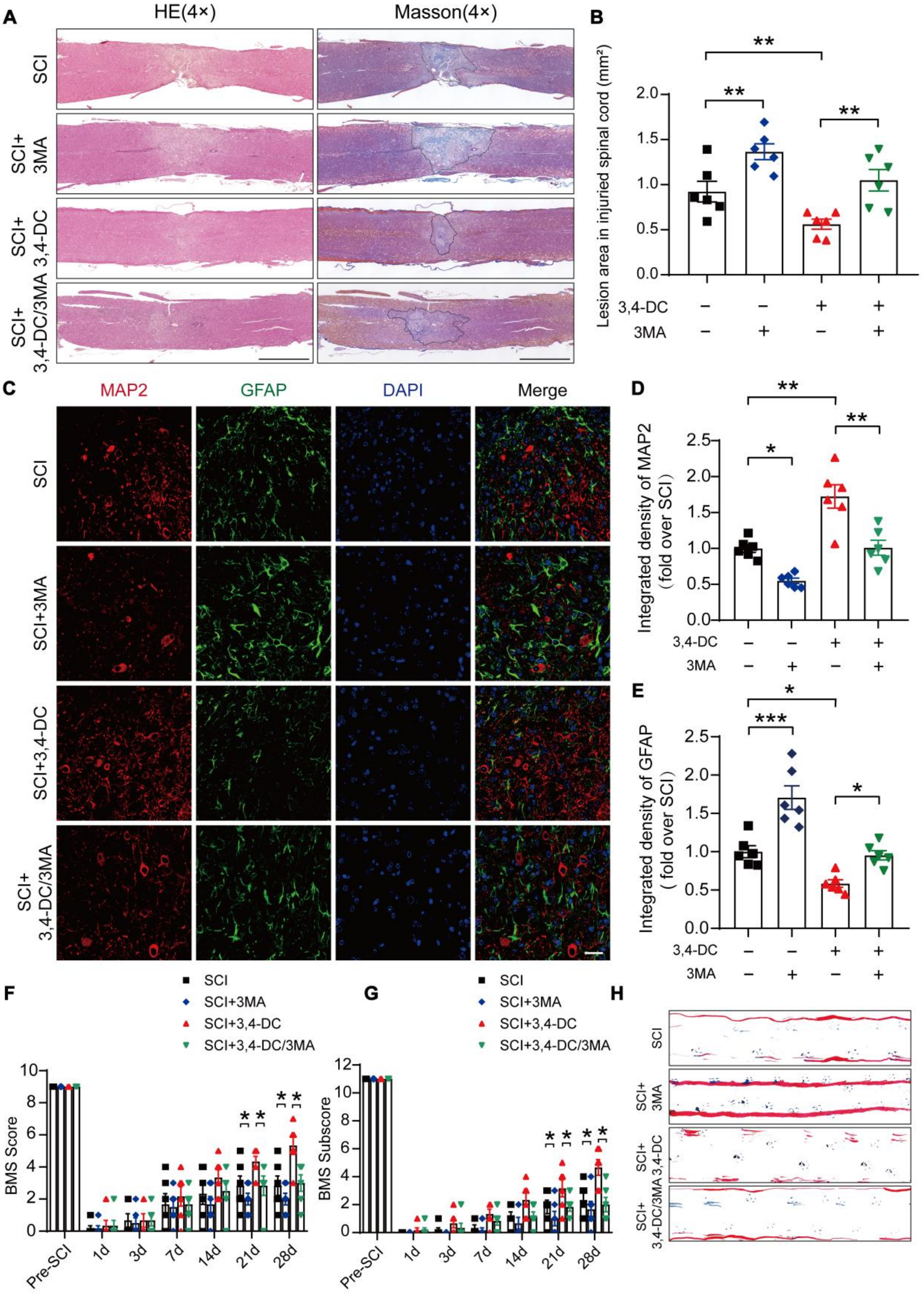

Figure S4. 3,4-DC enhances functional recovery by promoting autophagy. (A-B) The longitudinal sections of spinal cords from the indicated groups were analyzed by HE staining and Masson staining on day 28 after SCI; scale bar: 1000 uM.

Quantitative analysis of Masson positive lesions in the spinal cords of each group. **(C-E)** Representative images of immunofluorescent analysis of MAP2 (red) and GFAP (green) and DAPI (blue) in in sagittal sections of thoracic spinal cords on day 28 after SCI; scale bar: 100 μm. Quantitative analysis of MAP2 and GFAP immunofluorescence is shown in the graph on the right. **(F-G)** BMS score and subscore at indicated groups and time points. **(H)** Photos of mice footprints at 28 day. The data are presented as the means ± SEMs (n = 6 mice per group); $*P < 0.05$, $**P < 0.01$, and $***P < 0.001$ indicate significant differences; ns, not significant. Significance was calculated using a two-way ANOVA followed by Tukey's multiple comparison test.

Figure S5

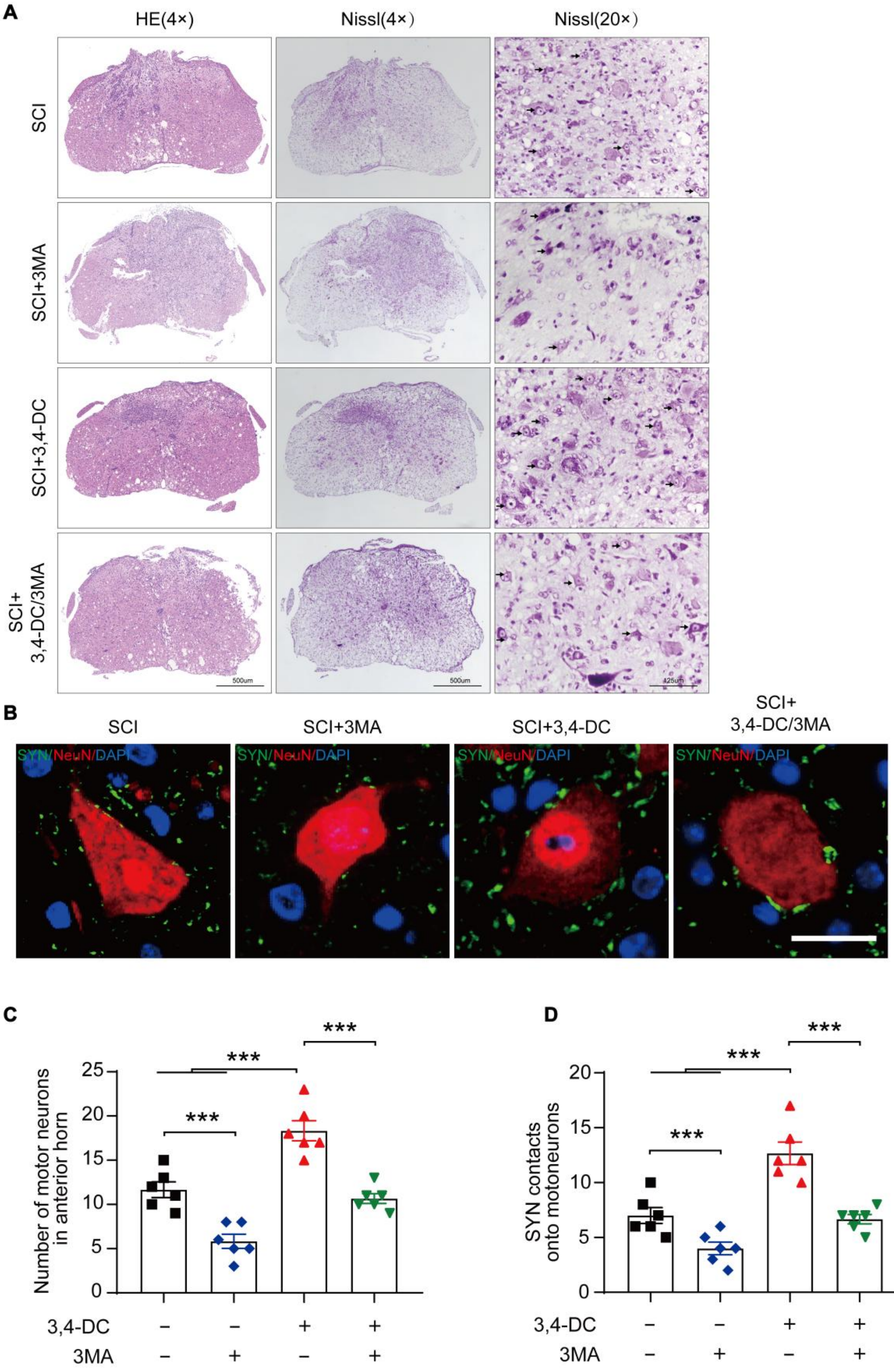

Figure S5. 3,4-DC inhibits motorneuron loss and promotes neuron-synaptic connections by promoting autophagy. (A) Transverse spinal cord sections from each group were analyzed by HE staining and Nissl staining on day 14 after SCI;

black arrows indicating the motoneurons; scale bar: 500 μm and 125 μm. **(B)** Representative images of spinal cord sections below the injury (T11-T12) and stained on day 28 after SCI with antibodies against SYN/NeuN; scale bar: 5 μm. **(C)** Quantitative analysis of Nissl positive motor neurons in the anterior horn of spinal cord from each group (the SCI, SCI+3MA, SCI+3,4-DC, and SCI+3,4-DC/3MA groups). **(D)** Relevant quantification of the number of synapses contacting onto motor neurons. The data are presented as the means ± SEMs (n = 6 mice per group); *P < 0.05, **P < 0.01, and ***P < 0.001 indicate significant differences; ns, not significant. Significance was calculated using a two-way ANOVA followed by Tukey's multiple comparison test.

Figure S6

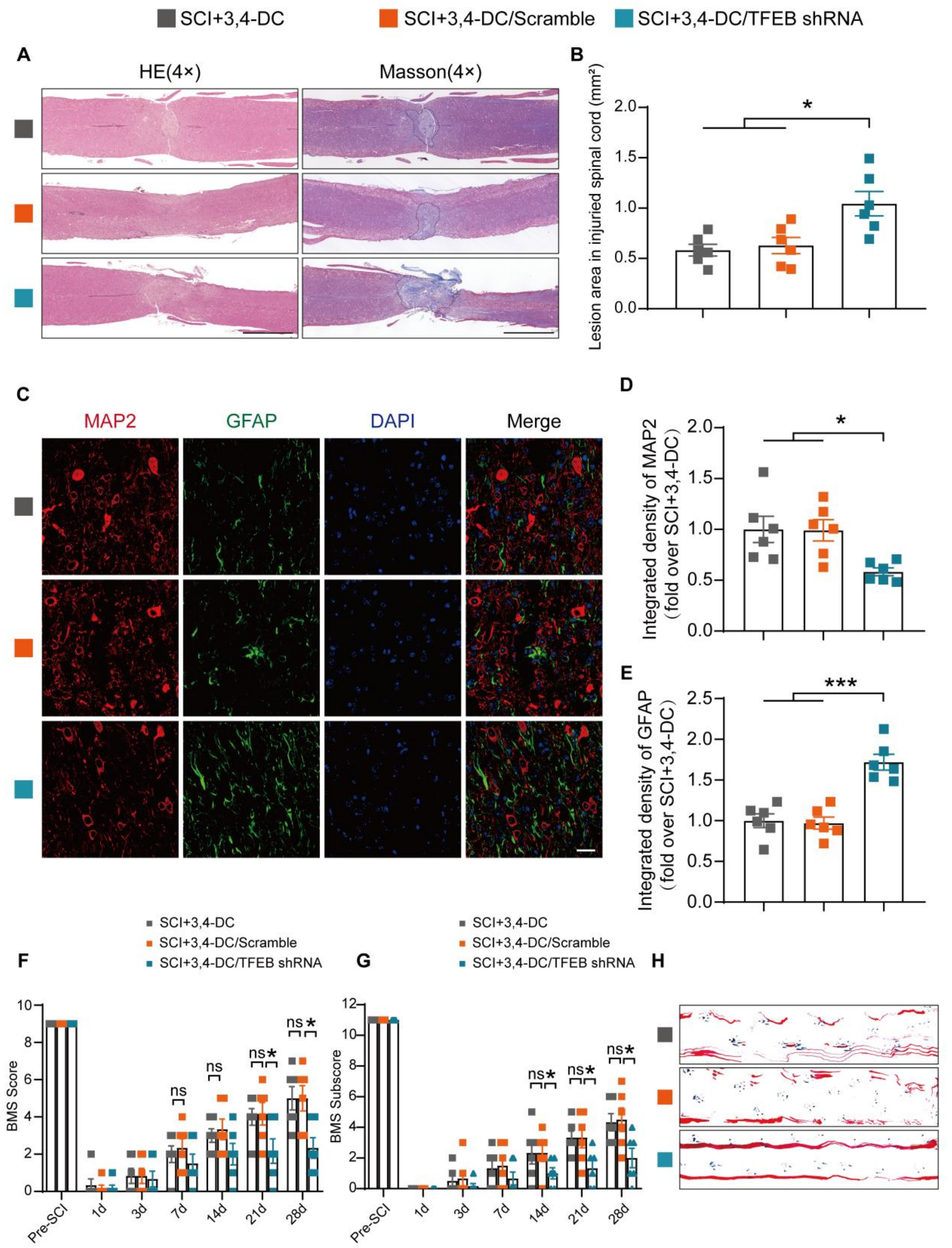

Figure S6. 3,4-DC enhances functional recovery by promoting TFEB. (A-B) The longitudinal sections of spinal cords from the indicated groups were analyzed by HE staining and Masson staining on day 28 after SCI; scale bar: 1000 μm. Quantitative

analysis of Masson positive lesions in the spinal cords of each group. **(C-E)** Representative images of immunofluorescent analysis of MAP2 (red) and GFAP (green), and DAPI (blue) in sagittal sections of thoracic spinal cords on day 28 after SCI; scale bar: 100 μm. Quantitative analysis of MAP2 and GFAP immunofluorescence is shown in the graph on the right. **(F-G)** BMS score and subscore at indicated groups and time points. **(H)** Photos of mice footprints analysis at day 28.The data are presented as the means ± SEMs (n = 6 mice per group); **P* < 0.05, ***P* < 0.01, and ****P* < 0.001 indicate significant differences; ns, not significant. Significance was calculated using a two-way ANOVA followed by Tukey's multiple comparison test.

Figure S7

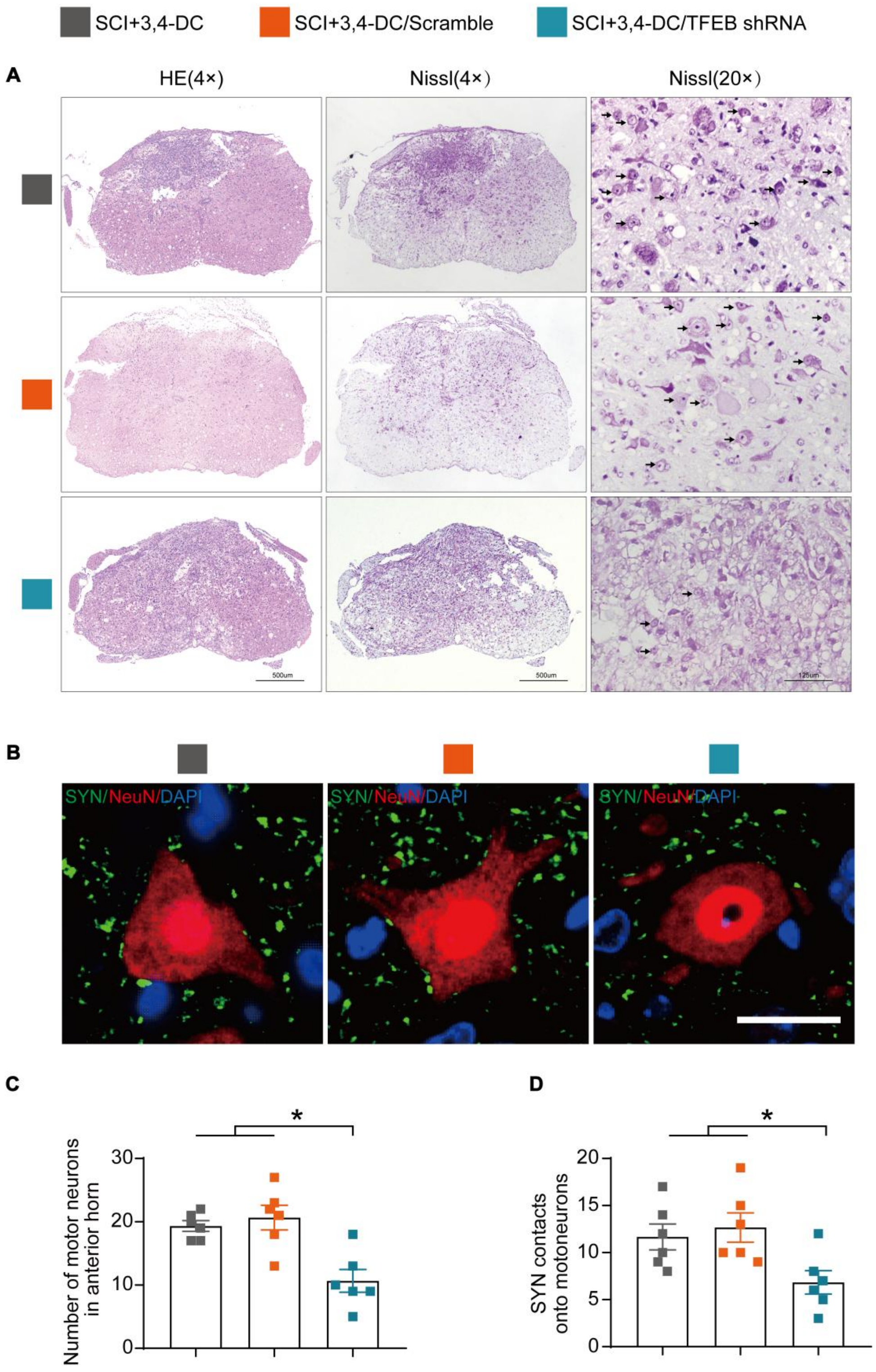

Figure S7. 3,4-DC inhibits motorneuron loss and promotes neuron-synaptic connections by promoting TFEB. (A) Transverse spinal cord sections from each group were analyzed by HE staining and Nissl staining on day 14 after SCI; black

arrows indicating the motoneurons; scale bar: 500 μm and 125 μm. **(B)** Representative images of spinal cord sections below the injury (T11-T12) and stained on day 28 after SCI with antibodies against SYN/NeuN; scale bar: 5 μm. **(C)** Quantitative analysis of Nissl positive motor neurons in the anterior horn of spinal cord from each group (the SCI+3,4-DC, SCI+3,4-DC/scrambled shRNA and SCI+3,4-DC/TFEB shRNA groups). **(D)** Quantification of the number of synapses contacting onto motor neurons. The data are presented as the means ± SEMs (n = 6 mice per group); *$P < 0.05$, **$P < 0.01$, and ***$P < 0.001$ indicate significant differences; ns, not significant. Significance was calculated using a two-way ANOVA followed by Tukey's multiple comparison test.

Figure S8

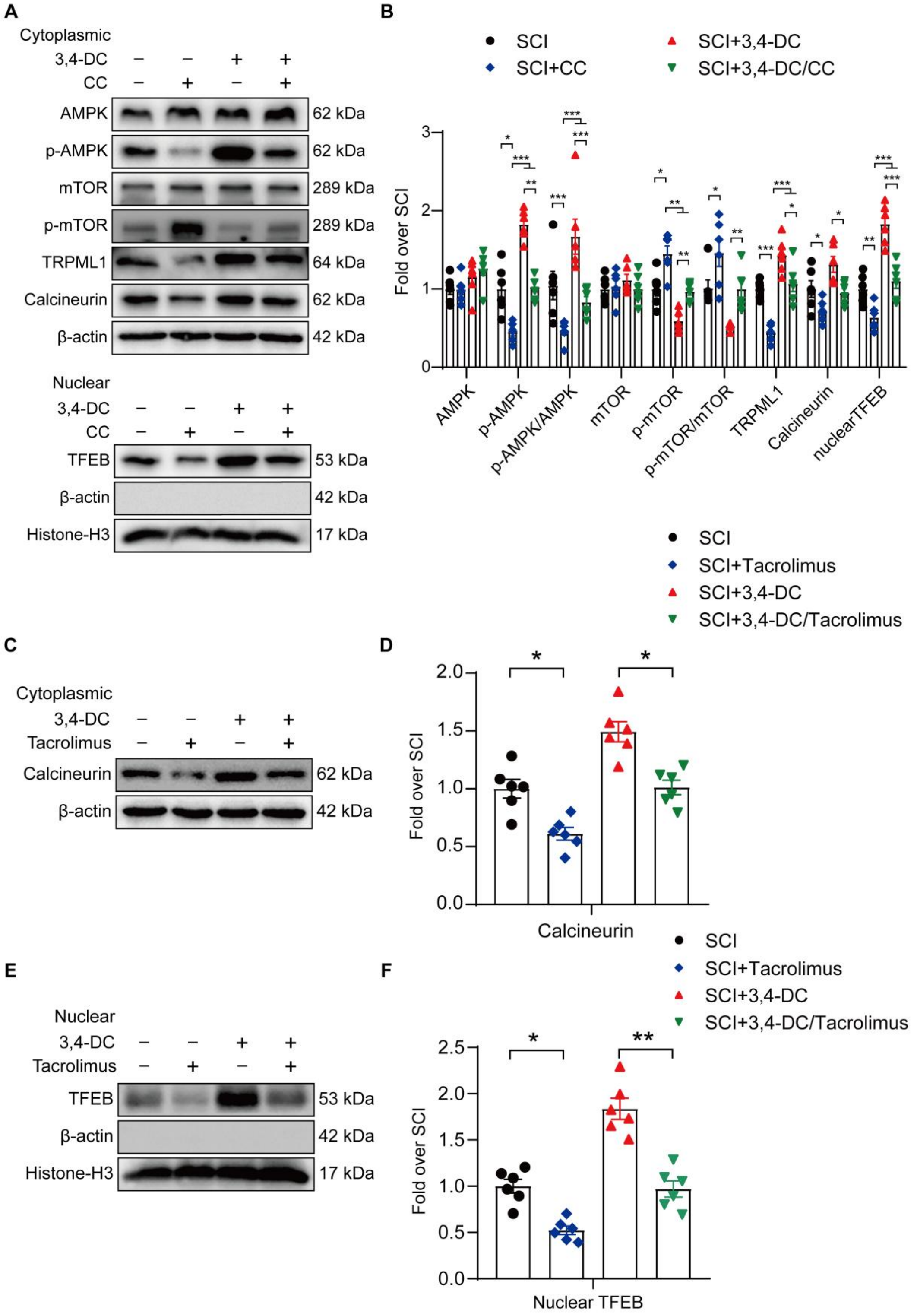

Figure S8. 3,4-DC activates TFEB may through the AMPK-TRPML1-calcineurin signaling pathway. (A-B) Protein levels of AMPK, p-AMPK, mTOR, p-mTOR, TRPML1, calcineurin and nuclear TFEB in the injured

spinal cord of all groups on day 3 after SCI (the SCI, SCI+CC, SCI+3,4-DC, and SCI+3,4-DC/CC groups). β-actin or histone H3 was utilized as the loading control. **(C-F)** Representative western blot analysis of Calcineurin and TFEB in the injured spinal cord lesion from SCI, SCI+Tacrolimus, SCI+3,4-DC, and SCI+3,4-DC/Tacrolimus groups. β-actin or Histone-H3 was used as controls. The graphs on the right show the summary data from western blots. The data are presented as the means ± SEMs (n = 6 mice per group); *$P < 0.05$, **$P < 0.01$, and ***$P < 0.001$ indicate significant differences; ns, not significant. Significance was calculated using a two-way ANOVA followed by Tukey's multiple comparison test.

Tables

Antibodies/Reagents	Source	Concentration for WB	Concentration for IF	Identifier
GFAP	Santa Cruz	/	1:200	cat# sc-33673
Beclin1	CST	1:1000	/	cat# 3738
NLRP3	CST	1:1000	/	cat# 15101
TMS1/ASC	CST	1:1000	/	cat# 67824
LC3	CST	1:1000	1:200	cat# 3868
β-actin	CST	1:1000	/	cat# 8457
cleaved caspase-8	CST	1:1000	/	cat# 8592
acetyl-α-tubulin	CST	1:1000	/	cat# 5335
α-tubulin	CST	1:1000	/	cat# 2125
AMPK	CST	1:1000	/	cat# 5832
p-AMPK	CST	1:1000	/	cat# 2531
mTOR	CST	1:1000	/	cat# 2983
p-mTOR	CST	1:1000	/	cat# 5536
VPS34	proteintech	1:1000	/	cat# 12452-1-AP
cathepsin D (CTSD)	proteintech	1:1000	/	cat# 21327-1-AP
caspase-8	proteintech	1:1000	/	cat# 13423-1-AP
TXNIP	proteintech	1:1000	/	cat# 18243-1-AP
caspase-1	proteintech	1:1000	1:200	cat# 22915-1-AP
histone H3	proteintech	1:1000	/	cat# 17168-1-AP
GAPDH	proteintech	1:4000	/	cat#

				10494-1-AP
RIPK1	Abcam	1:1000	1:200	cat# ab106393
RIPK3	Abcam	1:1000	1:200	cat# ab62344
p-MLKL	Abcam	1:1000	/	cat# ab196436
TRPML1/MG-2	Abcam	1:1000	/	cat# ab28508
NeuN	Abcam	/	1:500	cat# ab177487
Iba-1	Abcam	/	1:500	cat# ab283319
synaptophysin	Abcam	/	1:500	cat# ab32594
MAP2	Abcam	/	1:500	cat# ab32454
p62/SQSTM1	Abcam	1:1000	1:200	cat# ab56416
goat anti-rabbit IgG H&L (Alexa Fluor® 488)	Abcam	/	1:1000	cat# ab150077
goat anti-mouse IgG H&L (Alexa Fluor® 647)	Abcam	/	1:1000	cat# ab150115
Goat anti-mouse IgG (H+L)-HRP	Bioworld	/	1:500	cat# BS12478
Goat anti-rabbit IgG (H+L)-HRP	Bioworld	/	1:500	cat# BS13278
IL-1β	ABclonal	1:1000	/	cat# A11369
TFEB	ABclonal	1:1000	1:200	cat# A7311
calcineurin	ABclonal	1:1000	/	cat# A4346
MLKL	Affinity	1:1000	/	cat# DF7412
IL-18	Affinity	1:1000	/	cat# DF6252
p-RIPK1	Affinity	1:1000	/	cat# AF2398
p-RIPK3	Affinity	1:1000	/	cat# AF7443
GSDMD	Affinity	1:1000	1:200	cat# AF4013
p70S6K	HuaBio	1:1000	/	cat# ER31205

p-p70S6K	HuaBio	1:1000	/	cat# RT1456
3,4-Dimethoxychalcone	Extrasynthese	/	/	cat# ZES-1294
3-Methyladenine (3MA)	Sigma–Aldrich	/	/	cat# M9281
Compound C	Med Chem Express	/	/	cat# 866405-64-3
MHY1485	Med Chem Express	/	/	cat# HY-B0795
tacrolimus	Med Chem Express	/	/	cat# HY-13756
gentamicin sulfate	Med Chem Express	/	/	cat# HY-A0276

Table S1. Detailed description of antibodies and reagents used in this study.

Groups	Mice	Procedure	Treatments	Number
Sham	C57BL/6	Sham	/	30
SCI	C57BL/6	SCI	/	33
SCI+3,4-DC	C57BL/6	SCI	3,4-DC (200 mg/kg)	33
SCI+3MA	C57BL/6	SCI	3MA (15 mg/kg)	24
SCI+3,4-DC/3MA	C57BL/6	SCI	3,4-DC (200 mg/kg)+3MA (15 mg/kg)	24
SCI+3,4-DC/scrambled shRNA	C57BL/6	SCI	3,4-DC (200 mg/kg)+scrambled shRNA (5×10^9 genomic particles, 2 ul), *in situ* injection	24
SCI+3,4-DC/TFEB shRNA	C57BL/6	SCI	3,4-DC (200 mg/kg)+TFEB	24

			shRNA (5 × 10^9 genomic particles, 2 ul), *in situ* injection	
SCI+Compound C	C57BL/6	SCI	Compound C(1.5 mg/kg)	6
SCI+3,4-DC/Compound C	C57BL/6	SCI	3,4-DC (200 mg/kg)+Compound C(1.5 mg/kg)	6
SCI+MHY1485	C57BL/6	SCI	MHY1485 (2 mg/kg)	6
SCI+3,4-DC/MHY1485	C57BL/6	SCI	3,4-DC (200 mg/kg)+MHY1485 (2 mg/kg)	6
SCI+tacroliums	C57BL/6	SCI	tacrolimus (1 mg/kg)	6
SCI+3,4-DC/tacroliums	C57BL/6	SCI	3,4-DC (200 mg/kg)+tacrolimus (1 mg/kg)	6
SCI+3,4-DC	C57BL/6	SCI	3,4-DC (100 mg/kg)	6
SCI+3,4-DC	C57BL/6	SCI	3,4-DC (150 mg/kg)	6
SCI+3,4-DC	C57BL/6	SCI	3,4-DC (250 mg/kg)	6
Sham+3,4-DC	C57BL/6	Sham	3,4-DC (200 mg/kg)	30

Table S2. Animals, treatments and groups in the study.

Primer name	Primer sequences
Beclin1	*5'- AAACTGGACACGAGCTTC-3' (forward)*<br>*5'-CCTGGCGAGTTTCAATAAATG-3' (reverse)*
Vps34	*5'-CCTCTATGTGACTTGTCAAGTG-3' (forward)*<br>*5'-TGTACTAAACGCCTTGTAGGA-3' (reverse)*
Sqstm1	*5'-GATAGCCTTGGAGTCGGT-3' (forward)*<br>*5'-AAATGTGTCCAGTCATCGTC-3' (reverse)*

Ctsd	*5'-AGTCAAAGGCAAGAGGTATCA-3' (forward)*<br>*5'-TTGGCTGCAACAAATACGATTC-3' (reverse)*
Lc3	*5'-ATGGTGAGCGTCTCCACA-3' (forward)*<br>*5'-GTTTCTTGGGAGGCGTAG-3' (reverse)*
β-actin	*5'-GGCTCCTAGCACCATGAAGA-3' (forward)*<br>*5'-AGCTCAGTAACAGTCCGCC-3' (reverse)*

Table S3. Information of the primer sequences for qPCR.
